# Structure-Transport Relationships in Aqueous Fructose Solutions: A Combined Molecular Dynamics and NMR Diffusion Study

**DOI:** 10.1021/acs.jpcb.6c04242

**Published:** 2026-09-16

**Authors:** San Vo Vinh Phan, Xinyue Zhou, Gabriel D. Barbosa, Tran Thi Bao Le, Christopher M. A. Parlett, Xiaolei Fan, Carmine D’Agostino, Alberto Striolo

**Affiliations:** † School of Sustainable Chemical, Biological, and Materials Engineering, The University of Oklahoma, 100 E. Boyd Street, Norman, Oklahoma 73019, United States; ‡ Department of Chemical Engineering, 5292 The University of Manchester, Oxford Road, Manchester M13 9PL, United Kingdom; § Dipartimento di Ingegneria Civile, Chimica, Ambientale e dei Materiali (DICAM), Alma Mater Studiorum—Università di Bologna, Via Terracini, 28, 40131 Bologna, Italy; ∥ Materials Science and Engineering Program, The University of Oklahoma, Norman, Oklahoma 73019, United States

## Abstract

Fructose is widely used in the food industry and is proposed as a sustainable biomass feedstock for chemical manufacturing. Upon dissolution in water, it undergoes mutarotation into different tautomers, primarily β-d-fructopyranose (68%) and β-d-fructofuranose (22%), as well as into the linear keto d-fructose structure. Although present only in small amounts, the linear tautomer may alter diffusion within aqueous media and porous catalytic materials. To elucidate the structure-transport properties of aqueous solutions containing fructose, molecular dynamics (MD) simulations are combined with nuclear magnetic resonance (NMR) diffusion experiments. Classic MD simulations are conducted at atomistic resolution using three different force fields. The NMR diffusion experiments are conducted in deuterated water to differentiate water from sugar peaks in the experimental signal. When the OPLS-AA force field is implemented, the MD simulated diffusion coefficients, as well as bulk system densities, are consistent with experiments, indicating the atomistic models are realistic. Interrogating the simulation trajectories confirms that fructose molecules aggregate into clusters, which lowers the diffusion coefficients of both fructose and water at high fructose concentration. Modest differences are observed in the mobility of the two cyclic tautomers, even in the presence of the linear counterpart. Such insight into the structure and mobility of fructose in bulk aqueous solutions across a range of concentrations is paramount to understanding several industrial processes, for example those involved in catalytic biomass upgrading technologies.

## Graphical abstract

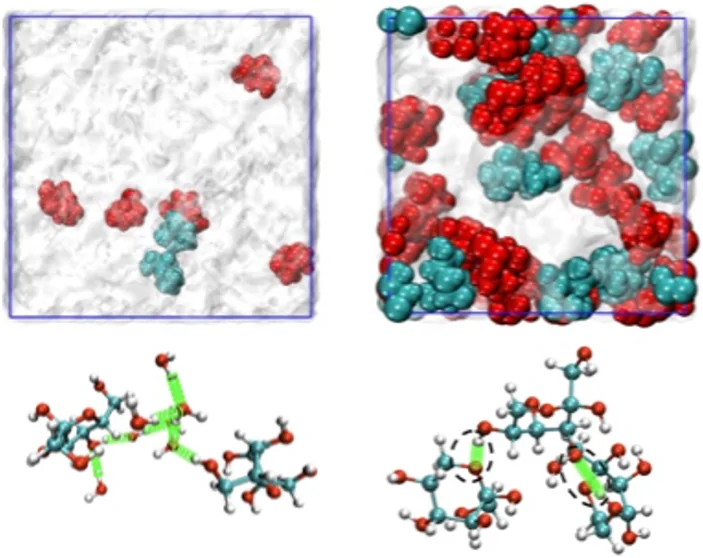

## 1 Introduction

Fructose is an abundant natural feedstock obtained from corn starch or through the hydrolysis of sucrose. It is widely used in the food industry, particularly in the form of high-fructose corn syrup (HFCS), which typically contains 45–55% fructose, whereas lignocellulosic biomass represents a non-edible feedstock. Fructose has also attracted growing interest as a renewable platform molecule for the production of chemicals.^1,2^ In particular, its conversion to 5-hydroxymethylfurfural (HMF) represents an important route toward sustainable chemicals manufacturing.^2–4^

Fructose is commonly processed, transported, and reacted in liquid media; thus, understanding its behavior in aqueous solutions is essential for developing reliable structure-transport relationships. Sugar mobility in bulk water is strongly affected by concentration and temperature. Of relevance to the development of structure-transport relations, Mahawanich et al.^5^ investigated the relationship between sugar mobility and perceived sweetness for sucrose, fructose, and glucose over a wide concentration range of 0.15–6.19 M using the pulsed-field gradient spin-echo (PGSE)-nuclear magnetic resonance (NMR) technique. Their results showed that fructose exhibits higher molecular mobility than both glucose and sucrose. Similarly, Ribeiro et al.^6^ reported mutual diffusion coefficients as obtained using the Taylor dispersion method for several aqueous sugar solutions, including sucrose, lactose, glucose, and fructose, across different temperature–concentration conditions. The findings confirmed that fructose consistently displays the highest mobility among the sugars studied. A key observation emerging from these studies is that fructose mobility decreases markedly as its concentration increases, a trend that has also been observed for other sugars by Mahawanich et al.^5^ and Ribeiro et al.^6^ This concentration-dependent slowdown reflects changes in the local liquid structure, presumably due to the association of sugar with other molecules in the system. However, the molecular mechanisms responsible for these transport trends remain insufficiently resolved. Establishing a detailed connection between solution structure and molecular mobility is important not only for fundamental understanding but also for optimizing processes involving fructose-rich aqueous media.

NMR spectroscopy provides a powerful experimental route for probing the structural and dynamic properties of aqueous carbohydrate systems.^7,8^ Specifically, pulse field gradient nuclear magnetic resonance (PFG-NMR), which was first demonstrated by Stejskal & Tanner,^9^ is a useful technique to investigate the translational mobility of nuclear spins, information that can be used to infer molecular diffusion. As a relevant example, Rampp et al.^10^ investigated aqueous carbohydrate solutions over a range of temperature and concentration conditions employing PGSE-NMR. They reached the general conclusion that at low concentration (*c* < 50% w/w), all four carbohydrates have nearly the same intra-diffusion coefficient at equal concentration and temperature. Similarly, several other studies have used NMR methods to quantify sugar diffusivities in water.^5,11,12^ Importantly, NMR-derived transport properties can be directly compared with atomistic molecular dynamics simulations, making the two approaches highly complementary. NMR provides experimentally grounded measurements of molecular mobility, whereas molecular dynamics (MD) can resolve the underlying molecular configurations and interactions that give rise to the observed transport behavior. MD simulations have been widely applied to systems of increasing complexity, from bulk gaseous mixtures^13^ to aqueous glucose solutions confined in zeolites.^14^ Considering fructose-containing systems, Pomata et al.^15^ used MD to examine how intermolecular and intramolecular interactions influence fructose aggregation in water. A central challenge in such simulations is the selection of an appropriate force field, without which it is impossible to secure reliable connections with experiments. Among other force fields,^16,17^ those suitable to study carbohydrates include CHARMM,^18–21^ GLYCAM06,^22^ and OPLS-AA.^23,24^

Here, we employ three representative carbohydrate force fields to simulate aqueous fructose systems across the concentration range of 0.11–4.85 M. The simulations primarily consider the two most abundant fructose tautomers in water, i.e., β-d-fructofuranose (FUR) and β-d-fructopyranose (PYR).^25–28^ The resulting simulated diffusion coefficients are benchmarked against literature PGSE-NMR data,^5,29^ and against new PFG-NMR measurements conducted in deuterated water, which enables clearer discrimination between fructose- and water-derived signals. Following validation, the MD simulations were analysed to elucidate the molecular structure of aqueous fructose solutions as a function of system composition and to assess whether mobility differences arise between fructose tautomers, including in the presence of a minor keto d-fructose (KETO) population and dissolved sodium chloride. To rationalize the observed experimental results, hydrogen-bond structures and residence time of selected paired configurations are quantified from the simulated trajectories. Together, the combined NMR-MD approach establishes a molecular-level structure-transport relationship for fructose in bulk aqueous solutions.

## 2 Methods

### 2.1 Experimental NMR Protocols

PFG-NMR experiments were performed using a Magritek SpinSolve benchtop NMR spectrometer operating at the ^1^H frequency of 43 MHz. The experiments were carried out using a diffusion probe capable of generating magnetic field gradient pulses up to 163 mT m^–1^. Fructose solutions of different concentrations were prepared in D_2_O in order to minimise the interference from the ^1^H signal of H_2_O on the measurement of fructose diffusion. Diffusion measurements were performed using the PGSE sequence.^9^ The sequence combines radiofrequency (RF) pulses with magnetic field gradients (*g*). The attenuation of the NMR signal as a function of gradient strength, *E*(*g*), is related to the diffusion coefficient (*D*) according to

1 $$\frac{E\left(g\right)}{E_{0}}=\exp\left[-D\gamma^{2}g^{2}\delta^{2}\left(\Delta -\frac{\delta }{3}\right)\right]$$

where *g* is the strength of the gradient pulse of duration δ. The value of δ was varied between 1.2 and 4 ms to ensure sufficient signal attenuation of the studied components; Δ is the time interval between the leading edges of the gradient pulses (set to 50 ms); and *D* the self-diffusion coefficient. The *b* term, *b* = γ^2^δ^2^ *g* ^2^(Δ – δ/3)­[s m^–2^], is often referred to as the *b*-factor.

### 2.2 Simulation Methods and Algorithms

#### 2.2.1 System Composition

Upon dissolution in water, fructose undergoes mutarotation and exists as several tautomeric forms. Among these, β-d-fructofuranose and β-d-fructopyranose are the dominant structures, with reported abundances of 68.2% and 22.4%, respectively.^25^ Whether these two tautomers exhibit different mobilities in aqueous solution remains unclear. Hence, β-d-fructofuranose (FUR) and β-d-fructopyranose (PYR) were selected for atomistic MD simulations. Unless otherwise stated, the simulated systems contained 30% FUR and 70% PYR. In additional simulations, keto d-fructose (KETO) was included at a composition of 4%, while the FUR and PYR fractions were adjusted to 28% and 68%, respectively. The fructose concentrations were selected based on the study of Pomata et al.^15^ The number of molecules used in each simulated system are listed in Tables 1, 2, and 3, and the molecular structures of FUR, PYR, and KETO are shown in Figure 1.

**Table 1:** Simulated Aq. Fructose Solution Compositions^a^

System number	Fructose concentration (M)	Fructose concentration (wt %)	*N*_PYR_	*N*_FUR_	*N*_SOL_
1	0.11	2%	5	2	3430
2	0.28	5%	5	2	1323
3	0.39	7%	5	2	931
4	1	17%	10	4	686
5	1.85	30%	21	9	672
6	3.39	51%	46	20	634
7	4.85	68%	116	50	792

a *N*_PYR_, *N*_FUR_, and *N*_SOL_ denote the number of PYR, FUR, and water molecules, respectively, included in the simulated systems.

**Table 2:** Simulated Aqueous Fructose System Compositions Containing KETO and NaCl^a^

System number	Fructose concentration (M)	NaCl concentration (M)	*N*_PYR_	*N*_FUR_	*N*_KETO_	*N*_NaCl_	*N*_SOL_
1	0.28	0	68	28	4	0	19,000
2		0.075	69	28	4	27	19,000
3		0.375	70	29	4	137	19,000
4		0.744	71	30	4	278	19,000
5	1.85	0	163	67	10	0	5600
6		0.075	163	67	10	10	5600
7		0.375	163	67	10	50	5600
8		0.744	163	67	10	100	5600

a *N*_PYR_, *N*_FUR_, *N*_KETO,_ *N*_SOL_, and *N*_NaCl_ denote the number of PYR, FUR, KETO, water, and NaCl molecules, respectively.

**Table 3:** Compositions of Simulated Aqueous Fructose Systems Containing NaCl But No KETO^a^

System number	Fructose concentration (M)	NaCl concentration (M)	*N*_PYR_	*N*_FUR_	*N*_NaCl_	*N*_SOL_
1	0.28	0	70	30	0	19,000
2		0.075	71	30	27	19,000
3		0.375	72	31	137	19,000
4		0.744	73	32	278	19,000
5	1.85	0	168	72	0	5600
6		0.075	168	72	10	5600
7		0.375	168	72	50	5600
8		0.744	168	72	100	5600

a The notation follows the one defined in Table 2.

**Figure 1:**
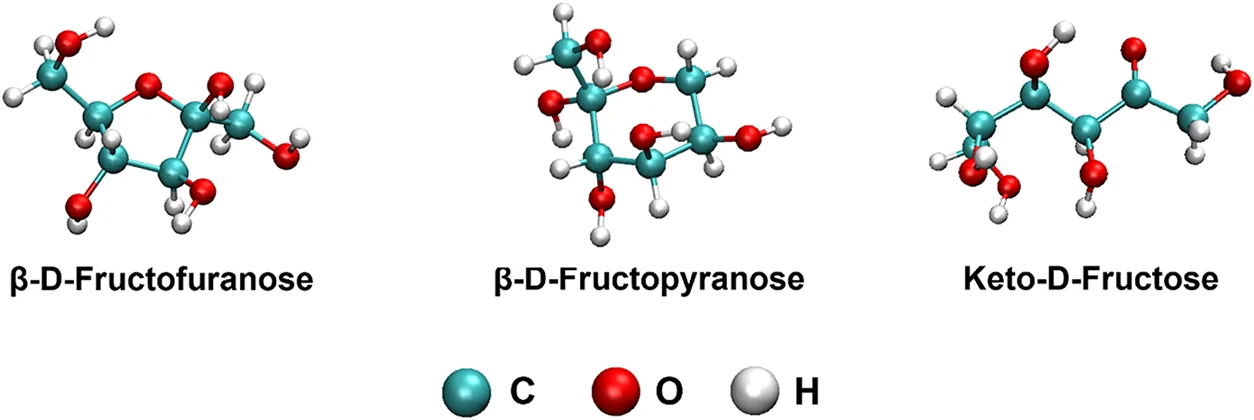
Molecular structures of the fructose tautomers considered in this study. In the molecular simulations presented, chemical reactions are not allowed.

#### 2.2.2 Force Fields

Three force fields commonly used for carbohydrate-containing systems were considered in this study: OPLS-AA, GLYCAM06, and CHARMM. A brief overview of their features and relevance to aqueous fructose simulations is provided in what follows.

The OPLS-AA force field, developed by Jorgensen et al., has been widely applied to simulate a wide range of chemical compounds.^23^ Building on the original formulation, Damm et al. introduced refined torsional parameters to improve the description of carbohydrates.^24^ Pomata et al. subsequently applied OPLS-AA to aqueous fructose solutions and reported good agreement with experimental NMR data, although their analysis focused primarily on water dynamics.^15^ OPLS-AA has also been used by Vlachos et al. to investigate solvent effects in fructose upgrading reactions,^30,31^ and by Zhu et al. to study the reaction pathways in fructose dehydration.^32^

The GLYCAM06 force field was developed specifically for carbohydrates, but it can be applied to more diverse classes of molecules such as hydrocarbons and alcohols.^22^ It has been applied to investigate interactions between fructose and organic solvents, producing results consistent with experiments.^33^ GLYCAM06 has also been used to simulate glucose-containing aqueous systems.^34^ In these studies, the self-diffusion coefficient of different sugars was found to decrease with increasing concentration; however, systematic benchmarking against experimental diffusion data remains limited.

The CHARMM General Force Field (CGenFF) extends the original CHARMM additive all-atom force field and is designed to describe a diverse range of biomolecules, including carbohydrate-related structures.^18–21,35^ Applications of CHARMM-based force fields have often focused on macromolecular systems, such as proteins and polysaccharides. By comparison, their application to small monosaccharides such as glucose and fructose is less extensive.

In this work, fructose parameters for the OPLS-AA were generated using the LigParGen web server.^36–38^ GLYCAM06 parameters were generated using the GLYCAM web server,^39^ while CHARMM-compatible parameters were obtained from the CGenFF web server.^35,40,41^ All force-field parameters were used directly without any additional reparameterization to describe the three fructose tautomers.

For all simulations, water was represented using the Extended Simple Point Charge (SPC/E) model.^42^ This model was selected because it provides a reliable description of bulk liquid water structure and self-diffusion, with reported values in good agreement with experimental data.^43,44^

Nonbonded interactions were described using electrostatic and dispersive contributions. Electrostatic interactions were modeled using the Coulombic potential, while dispersive interactions were represented using the 12–6 Lennard–Jones potential. The Lennard–Jones and Coulomb cutoffs were set at 11 Å for OPLS-AA and 12 Å for GLYCAM06, respectively. For CHARMM, the Lennard–Jones and Coulomb cutoffs were set to 10 Å and 11 Å, respectively. The Particle–Mesh Ewald (PME) method^45^ was used to correct long-range electrostatic interactions, with a PME order of 4 and a grid spacing of 1.6 Å.

#### 2.2.3 Algorithms

The MD simulations were carried out using the GROMACS software package (version 2023.3).^46–48^ The leapfrog algorithm^49^ with a time step of 2 fs was used to integrate the equations of motion. Periodic boundary conditions were applied in all three spatial directions. Temperature was maintained constant by the velocity-rescale thermostat^50^ with a coupling time of 2 ps, while pressure was constrained using the Parrinello–Rahman barostat^51^ with a coupling time of 10 ps. All bond lengths were constrained using the LINCS algorithm.^52^ Initial configurations were generated using PackMol.^53^

Each system was constructed in a cubic box with lateral dimensions of 8 nm. The fructose solutions were first relaxed via energy minimization using the steepest-descent method. The simulations were then conducted in the NPT ensemble (constant number of particles, pressure, and temperature) at 300 K and 1 bar for 40 ns. Equilibration was confirmed by achieving stable densities. The production run was then performed in the canonical ensemble (NVT, constant number of particles, volume, and temperature) for 100 ns to determine the diffusion coefficients and to analyze the structure of the various systems. For each concentration, four independent replicas were considered to minimize statistical uncertainty and mitigate data bias.^54^

From the simulated trajectories obtained during the production phase, several system properties were quantified: diffusion coefficients, hydrogen-bond networks, and various residence times. The diffusion coefficients (*D*) were extracted from the mean square displacements (MSD) profiles using the Einstein relations^55,56^

2 $$D=\frac{1}{2d}\lim_{t\to \infty }\frac{d}{dt}\left[\left\langle \frac{1}{N}\sum_{i=1}^{N}\left[r_{i}\left(t\right)-r_{i}\left(t_{0}\right)\right]\right\rangle \right]$$

where *d* is the dimensionality of the system, *r*_*i*_(*t*) and *r*_*i*_(*t*_0_) are the molecular positions at time *t* and *t*_0_, respectively, and *N* is the total number of molecules considered. As bulk systems are investigated in this study, *d* equals 3. The MSD plots obtained during the analysis of the simulated trajectories are shown in Figure S1 for each force field.

When evaluating the diffusion coefficients via MD, it is critical to consider finite simulation box-size effects, which result from several factors such as Coulombic and hydrodynamic interactions.^57,58^ As periodic boundary conditions are applied in all three directions, the computed diffusivity becomes inherently susceptible to finite-size effects.^54,56,59,60^ To correct for these biases, the limit at infinite-system-size for the diffusion coefficients at each concentration was obtained by the approach proposed by Maginn et al.,^54^ where the diffusion coefficient is evaluated as a function of the reciprocal box length (1/*L*). Size-effect corrections were applied as illustrated in Figures S2–S5. The limits to infinite-system diffusion coefficients were determined by extrapolating the results obtained from four independent system sizes, with the results for each size derived from four independent simulations.

The formation of a hydrogen bond between fructose molecules was identified via the following geometrical criterion:^15,61–63^ (i) the distance between donor and acceptor is less than 3.5 Å and (ii) the donor-hydrogen-acceptor angle must be greater than 150°. The same criteria were applied to characterize hydrogen bonds between fructose and water, as well as among water molecules. The hydrogen bond lifetime was calculated via the intermittent hydrogen bond autocorrelation function defined as follows^64^

3 $$C_{\mathrm{HB}}=\left\langle \frac{h_{\mathrm{ij}}\left(t_{0}\right)h_{\mathrm{ij}}\left(t_{0}+t\right)}{h_{\mathrm{ij}}{\left(t_{0}\right)}^{2}}\right\rangle$$

In eq 3, *h_ij_* equals 1 if a hydrogen bond exists between molecules *i* and *j*, and 0 otherwise.

## 3 Results

### 3.1 Validation of the Simulation Models: Density and Diffusion Coefficients

The reliability of the simulation models was first assessed by comparing the predicted densities of aqueous fructose solutions with experimental measurements. The simulated densities obtained in this work are reported in Table 4. In these simulations, the fructose tautomeric composition was fixed at a FUR/PYR ratio of 30:70. For all force fields, the solution density increases monotonically with fructose concentration. The corresponding concentration-dependent trends are shown in Figure S6. Although all force fields reproduce the same qualitative behavior, systematic differences are observed. The CHARMM force field yields the lowest densities across the concentration range, whereas GLYCAM06 gives the highest ones. The densities predicted by OPLS-AA are close to those obtained with GLYCAM06, although generally slightly lower. The experimentally measured densities of fructose solutions in both H_2_O and D_2_O, reported in Table 5, also increase monotonically with increasing fructose concentration, in good qualitative agreement with the simulations. Quantitatively, however, differences between the force fields become apparent. At higher fructose concentrations, ≥3.39 M, OPLS-AA and GLYCAM06 generally overestimate the experimental densities measured in H_2_O. In contrast, CHARMM slightly underestimates the experimental densities over the full concentration range. Overall, it is concluded that the force fields yield a reasonable description of the experimental systems in terms of density as a function of concentration.

**Table 4:** Simulated Densities of Aqueous Fructose Solutions, Calculated from the Average Results over Four Independent Simulations for Each System^a^

		Density (kg m^–3^)		
Fructose Concentration (M)	Simulation box length (Å)	OPLS-AA	GLYCAM06	CHARMM
0.11	38.89 ± 0.01	1004.7 ± 0.3	1005.7 ± 0.4	1000.4 ± 1.2
0.28	34.45 ± 0.03	1015.3 ± 0.8	1017.4 ± 0.5	1007.3 ± 0.5
0.39	30.75 ± 0.02	1023.2 ± 0.7	1025.2 ± 0.6	1013.8 ± 0.6
1	28.50 ± 0.01	1060.0 ± 0.8	1066.1 ± 0.7	1047.3 ± 0.7
1.85	29.72 ± 0.04	1115.8 ± 0.8	1127.5 ± 1.4	1098.8 ± 0.7
3.39	31.85 ± 0.03	1205.8 ± 0.9	1228.0 ± 0.6	1179.6 ± 1.5
4.85	38.48 ± 0.03	1288 ± 1	1321.1 ± 0.6	1254.3 ± 0.5

a All simulations were conducted at 300 K and 1 atm.

**Table 5:** Densities of Aqueous Fructose Solutions at Different Concentrations in H_2_O and D_2_O under Ambient Conditions (298 K, 1 atm), Measured Based on PFG-NMR Experimental Data

	Density (kg m^–3^)	
Fructose Concentration (M)	In H_2_O	In D_2_O
0.11	1017.1 ± 8.5	1115.9 ± 7.7
0.28	1019.7 ± 0.7	1126.7 ± 5.2
0.39	1035.4 ± 2.8	1132.8 ± 4.6
1	1088.4 ± 13.7	1153.2 ± 6.2
1.85	1127.8 ± 5.4	1180.9 ± 10.2
3.39	1202.4 ± 11.7	1242.5 ± 14.0
4.85	1229.4 ± 8.8	1310.2 ±7.5

Having established that the simulated densities reproduce the experimental trends reasonably well, the transport properties were next evaluated. As described in Section 2, the diffusion coefficients were extracted from MSD profiles for all the simulated systems using eq 2. The corresponding results are shown in Figure S7. Literature NMR data reported by Mahawanich et al.^5^ were first used as a benchmark to validate the simulation results. When the analysis was performed using the simulation box size listed in Table 4, the OPLS-AA and CHARMM force fields gave diffusion coefficients that were in reasonable agreement with the experimental data. In contrast, GLYCAM06 showed good agreement only at low sugar concentrations; at higher concentrations, the deviation from experiment became somewhat pronounced. Because diffusion coefficients obtained from MD simulations can be affected by the finite simulation box size, size-effect corrections were considered. For CHARMM, applying such corrections would likely lead to a substantial overestimation of the experimental diffusion coefficients. Therefore, additional simulations were conducted only for OPLS-AA and GLYCAM06 force fields to determine diffusion coefficients extrapolated to the infinite-system-size limit.

The corresponding results are compared with literature experimental data in Figure 2. At low fructose concentration, both simulation models predict faster fructose diffusion than that measured by the literature NMR data reported by Mahawanich et al.;^5^ the latter experimental data were obtained in water at 298 K. For GLYCAM06, the diffusion coefficient decreases sharply with increasing fructose concentration, reaching very low values at high concentrations. This suggests strong fructose-fructose association within this force-field description, leading to substantially hindered molecular motion. Such a pronounced slowdown is not consistent with the trend observed experimentally, indicating that GLYCAM06 may not be suitable for describing the aqueous fructose systems considered in this study. In contrast, the OPLS-AA force field reproduces the experimental concentration-dependent trend reasonably well at fructose concentrations above ∼ 1.8 M. However, at lower fructose concentrations, the simulated diffusion coefficients remain significantly higher than the literature NMR values. This discrepancy motivated the acquisition of new NMR measurements.

**Figure 2:**
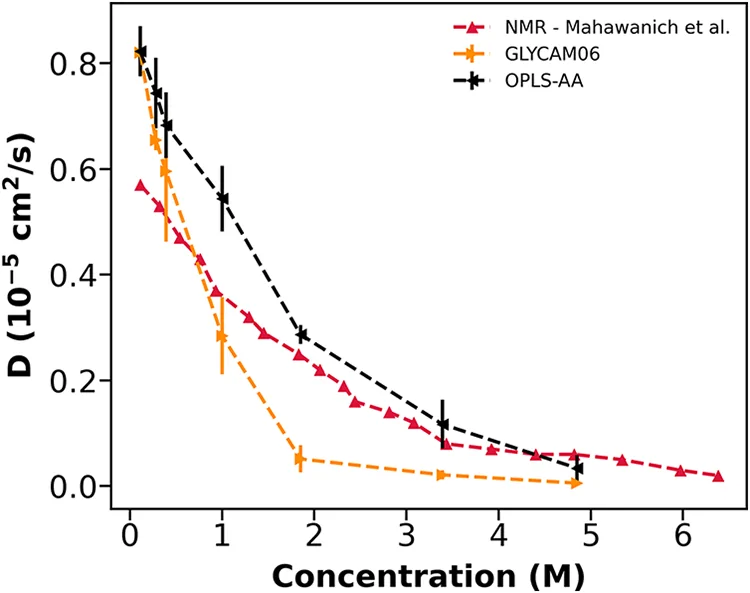
Fructose diffusion coefficients in aqueous solutions as a function of fructose concentration. The simulated values are extrapolated at the limit for an infinite simulation box size, implementing the GLYCAM06 and the OPLS-AA force fields. The simulation data were obtained at 300 K. Literature NMR data measured in water at 298 K are included for comparison.^5^ The error bars represent the 95% confidence interval as obtained using Student’s *t*-distribution.

### 3.2 PFG-NMR Measurements and Selection of the Force Field

To resolve the discrepancy between simulated diffusion coefficients and the literature experimental data shown in Figure 2, which were conducted in H_2_O, PFG-NMR measurements were performed using deuterated water. The resulting experimental diffusion coefficients are shown in Figure 3. For completeness, the corresponding pulsed gradient stimulated echo (PGSTE) log attenuation plots (Figure S8). In Figure 3, the new experimental data are compared to the simulation results obtained with the OPLS-AA force field. Excellent agreement is observed, especially at low fructose concentrations. At higher fructose concentrations, the simulations slightly underpredict the diffusion coefficients, but within statistical uncertainty. This may indicate that the previous measurements were affected by partial overlap between water and fructose signals.

**Figure 3:**
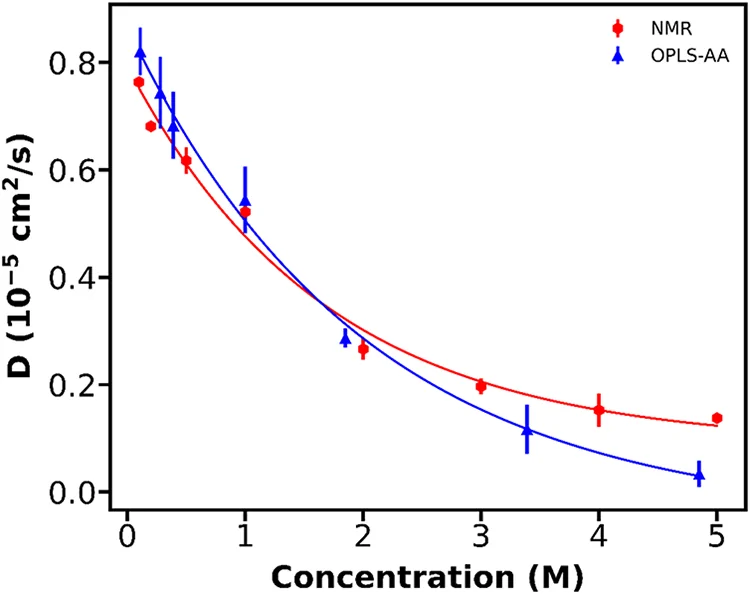
Fructose diffusion coefficient as a function of concentration in aqueous solutions. Experimental PFG-NMR data measured in deuterated water at 301 K (red); simulated values obtained using the OPLS-AA force field at 300 K (blue). Fitted blue and red continuous lines are included for visual guidance of the modeling and experimental to guide the eye, respectively. Detailed numerical values with 95% confidence intervals obtained using Student’s *t*-distribution are provided in Table S1.

Based on the density comparisons in Tables 4 and 5 and the fructose diffusion coefficients in Figure 3, OPLS-AA was identified as the most suitable force field among those considered in this study for modeling aqueous fructose solutions.

Using the OPLS-AA force field, the mobility of each tautomer in solution (FUR and PYR) was analyzed within the systems simulated. As shown in Figure S9, no significant difference was observed in the mobility of the tautomers. Of note, there is a notable lack of literature experimental studies directly comparing the mobility of the tautomers, as mutarotation between furanose and pyranose occurs very rapidly.^65^

In the remainder of the manuscript, the OPLS-AA force field was implemented to further test: structural properties as a function of sugar concentration; water diffusion coefficient; diffusion of different sugars; the effect of KETO; the effect of salts.

### 3.3 Molecular Structure-Transport Relationships

Having validated the OPLS-AA force field against experimental density and diffusion data, molecular simulations were used to interrogate the molecular structure of aqueous fructose solutions and to identify the origins of the observed concentration-dependent transport behaviour. Figure 4 depicts representative simulation snapshots at three fructose concentrations. Under dilute conditions (Figure 4A), fructose molecules are dispersed and interact predominantly with the surrounding water molecules, exhibiting only limited fructose–fructose association. This molecular environment is consistent with the relatively high fructose diffusion coefficients (Figure 3). As the concentration increases to 1.85 M (Figure 4B), fructose molecules associate, forming aggregates. These aggregates extend into clusters at the highly concentrated conditions considered (4.85 M, Figure 4C). The formation of such clusters restricts molecular motion, thereby contributing to the marked decrease in fructose diffusivity. Fructose molecules that are not directly incorporated into clusters may still experience constrained motion because the surrounding solution becomes increasingly crowded and structurally heterogeneous. These observations are consistent with the reduced diffusion coefficients reported in Figure 3. Similar aggregation behaviour has been reported previously by Pomata et al.^15^ and Sonoda et al.,^63^ who analyzed the size distribution of fructose aggregates and reported a sharp transition to a percolated cluster at concentrations between 3.5 and 3.8 M. The extended fructose aggregates observed in Figure 4B,C may also hinder the mobility of water molecules, an effect examined using both simulations and experiments in Section 3.5.

**Figure 4:**
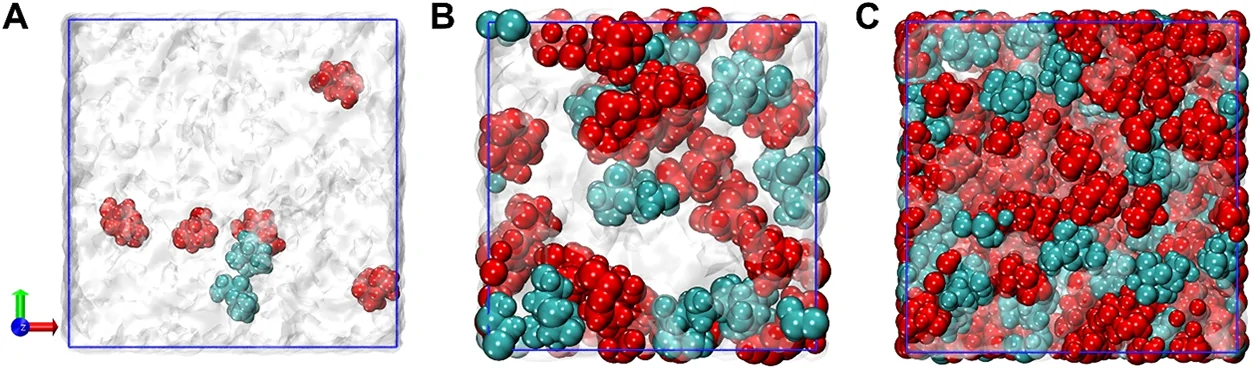
Simulation snapshots illustrating the system structure as obtained during the simulation of aqueous fructose solutions using the OPLS-AA force field. From left to right, the fructose concentration increases from 0.11 M (A) to 1.85 M (B) and 4.85 M (C). Red molecules represent PYR; cyan molecules represent FUR. Water is depicted as a transparent medium.

The snapshots in Figure 4 suggest that preferential fructose-fructose interactions play an important role in driving aggregation. To quantify these interactions, hydrogen-bond analyses were performed for fructose-fructose, fructose-water, and water–water pairs. The average number of hydrogen bonds is depicted in Figures 5 and 6. Notably, hydrogen bonds between FUR and PYR tautomers are rarely observed in the simulated trajectories. Instead, hydrogen bonding occurs predominantly between molecules of the same tautomeric type (i.e., FUR-FUR and PYR–PYR). Further, FUR molecules preferentially form hydrogen bonds with other FUR molecules rather than with water, whereas PYR molecules show a stronger tendency to hydrogen-bond with surrounding water molecules. The concentration-dependent variation in hydrogen bonding observed in our simulations is consistent with the trend reported by Pomota et al.^15^ in their investigation of water dynamics in carbohydrate solutions.

**Figure 5:**
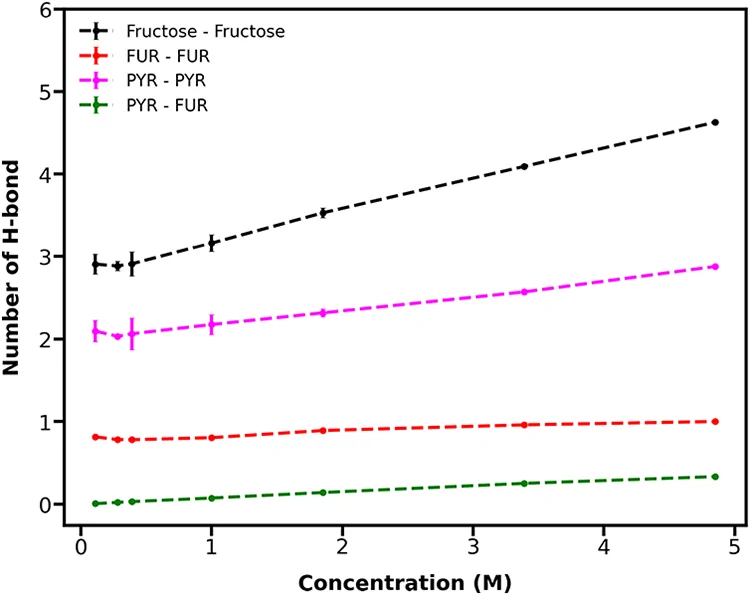
Average number of hydrogen bonds formed between fructose molecules at various concentrations extracted from the simulated trajectories. All values were normalized by the number of fructose molecules in the system. All simulations were conducted at 300 K. The error bars represent the 95% confidence interval as obtained using Student’s t-distribution.

**Figure 6:**
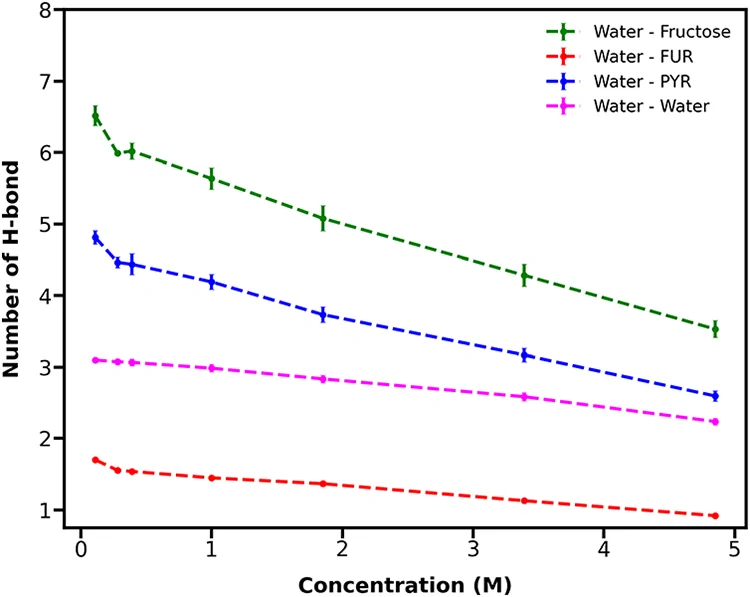
Average number of hydrogen bonds between water and fructose molecules at various concentrations as extracted from the simulated trajectories. All values were normalized by the total number of fructose molecules, except for water–water hydrogen bonds, which were normalized by the number of water molecules. All simulations were conducted at 300 K. The error bars represent the 95% confidence interval obtained using Student’s *t*-distribution.

The simulation results suggest that the hydrogen-bonded netw is highly dependent on fructose concentration. At low concentrations, fructose molecules primarily form hydrogen bonds with surrounding water molecules, as shown in Figure 6. Snapshots such as those in Figure 4 show that at these conditions, fructose molecules prefer to be surrounded by water. This observation is consistent with the results of Mathlouthi et al.,^66^ who reported Raman spectral shifts indicating extensive hydrogen bonding between fructose and water at around 30% (w/w), corresponding to ∼1.85 M in the present study. At higher concentrations, above 50% (w/w) (∼3.45 M), hydrogen bonding is increasingly dominated by fructose-fructose interactions. The results suggest that fructose molecules act as structure-breakers in the dilute regime, disrupting the network of hydrogen bonds between water molecules. As the solution is more concentrated, fructose behaves increasingly as structure-maker, promoting solute–solute association and the formation of water-rich domains or clusters.^67,68^ When the concentration approaches 4.85 M, fructose molecules engage in both intra- and intermolecular hydrogen bonding, as illustrated in Figures 7 and 8. In these structures, intermolecular O–H···O hydrogen bonds occur between the hydroxyl oxygen of one fructose molecule and the hydroxyl hydrogen of a neighboring molecule. Intramolecular hydrogen bonds are formed through internal O–H···O interactions between the hydroxyl groups within the fructose ring. The simulation trajectories also show that intermolecular hydrogen bonds can also form between FUR and PYR molecules via O–H···O interactions. These interactions further promote the aggregation of fructose into aggregates and clusters, thereby reducing the mobility of fructose.

**Figure 7:**
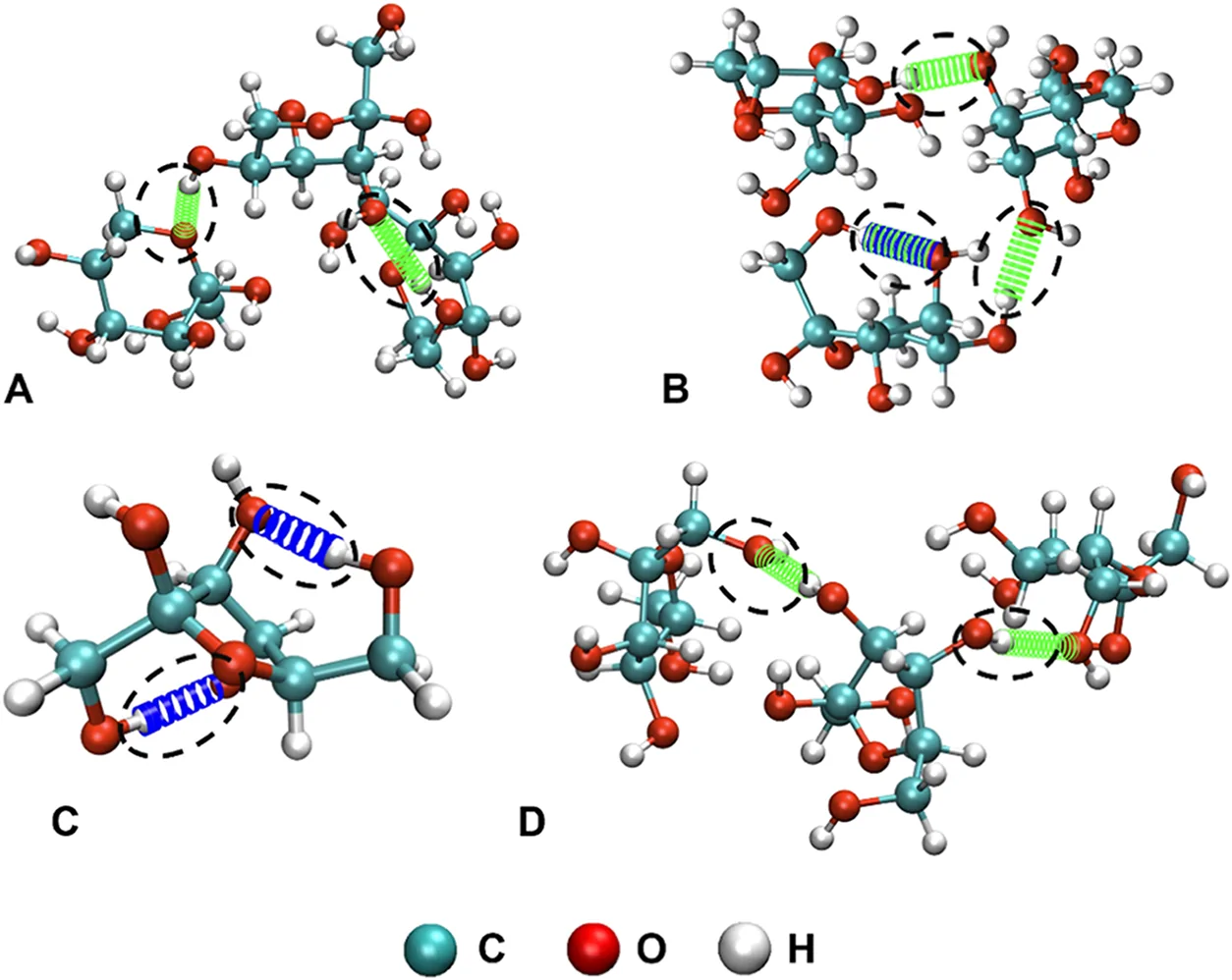
Simulation snapshots illustrating intermolecular and intramolecular hydrogen bonds formed between fructose molecules. (A) Hydrogen-bonding structure between FUR and PYR molecules. (B) Hydrogen-bonded structure between PYR molecules. (C) Intramolecular hydrogen bonds within the FUR molecule. (D) Hydrogen-bonded structure between FUR molecules. Green springs indicate intermolecular hydrogen bonds, while blue springs indicate intramolecular hydrogen bonds. The snapshots were generated using the VMD software for the system simulated at 4.85 M.

**Figure 8:**
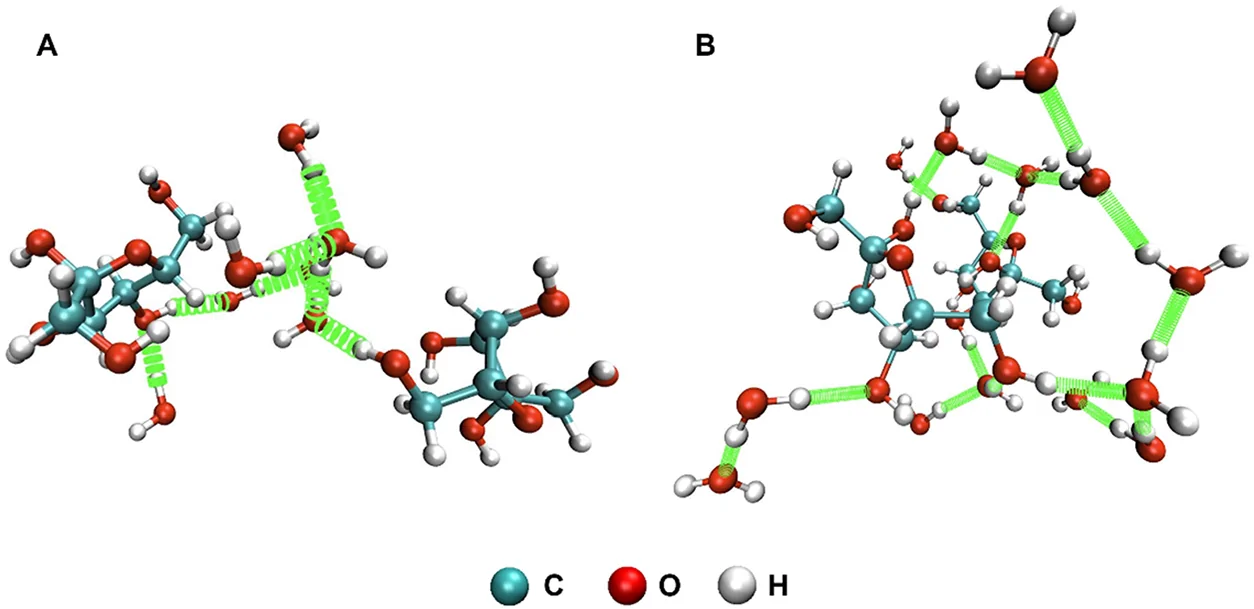
Simulation snapshots illustrating the hydrogen-bond network formed between fructose and water molecules. (A) Hydrogen bonds between FUR and water. (B) Hydrogen bonds between PYR and water. Green springs indicate intermolecular hydrogen bonds, while blue springs indicate intramolecular hydrogen bonds. The snapshots were generated using the software VMD for the system simulated at 4.85 M.

The lifetime of hydrogen bonds between fructose–fructose, fructose–water, and water–water molecules was further examined using the hydrogen-bond autocorrelation function. The results shown in Figure 9 represent averages from four independent calculations. Across the three concentrations considered (0.11, 1.85, and 4.85 M), the autocorrelation functions for fructose–water and water–water interactions decay more rapidly than those representing fructose–fructose interactions. This indicates that, at low concentration, fructose–water hydrogen bonds have the shortest lifetimes and are less stable than both fructose–fructose and water–water hydrogen bonds. As the concentration increases, the hydrogen-bond autocorrelation functions decay more slowly, indicating an increase in the average lifetime of the hydrogne bonds. At the highest concentration investigated (4.85 M), the hydrogen bonds between fructose molecules become significantly more persistent. A similar trend is observed for fructose–water interactions, whose hydrogen-bond lifetimes also increase with concentration. In contrast, the stability of water–water hydrogen bonds decrease at high fructose concentrations, reflecting the disruption of the water hydrogen-bond network by the increasing number of fructose molecules.

**Figure 9:**
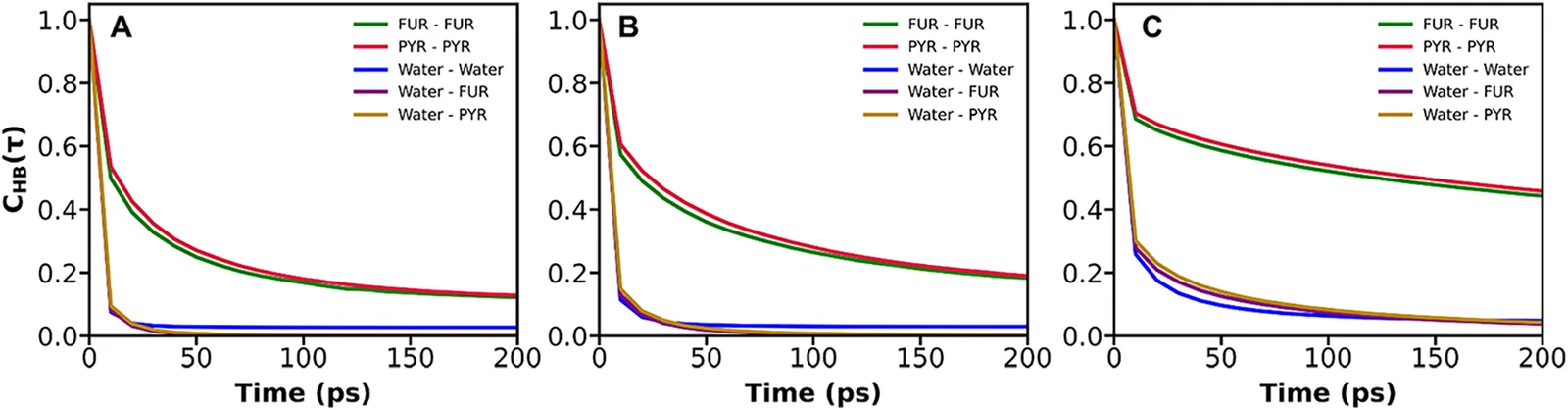
Hydrogen bond–hydrogen bond autocorrelation function obtained from simulations conducted for fructose solution at various concentrations. From left to right, the fructose concentration increases from 0.11 M (A) to 1.85 M (B) and 4.85 M (C). The simulation data were obtained at 300 K.

### 3.4 Effect of Additives

In addition to the two dominant cyclic tautomers of fructose, PYR and FUR, the linear KETO configuration may also exist in aqueous solutions due to the rapid interconversion among tautomers.^25–28^ Despite its low amount in aqueous solutions, this acyclic form is considered a key reactive specie in the dehydration of fructose through the acyclic mechanism.^69,70^ Further, although the KETO tautomer only constitutes ∼0.5% of the fructose molecules in aqueous systems,^25–28^ Wang et al.^70^ suggested that it is the primary reactant in the acyclic mechanism responsible for the dehydration of fructose into 5-hydroxymethylfurfural within H-ZSM-5 zeolite. Therefore, investigating its influence on the mobility of the other tautomers, as well as its own mobility is crucially important. Because of its linear structure, KETO may diffuse more readily than FUR and PYR and may facilitate the mobility of other molecules. Both latter possibilities were tested by conducting MD simulations for systems containing FUR, PYR, and KETO. In these simulations, the size-effect correction is not applied for brevity as the goal is to identify trends and changes in transport mechanisms. Although finite-size effects cannot be discarded with certainty, the analytical correction factor proposed by Yeh and Hummer suggests that the the box sizes of the 0.28 and 1.85 M systems (8.4 and 6.01 nm, respectively) are large enough to minimize spurious finite-size effects because the high concentration of fructose and salt increases viscosity.^59^ The MSD results are shown in Figures S10 and S11. The diffusivity results are shown in Figure 10 and reveal that the linear KETO tautomer consistently exhibits a higher diffusion coefficient compared to the cyclic forms, as expected because of its linear structure. However, the presence of KETO exerts a negligible influence on the transport of the dominant cyclic isomers when statistical errors are considered.

**Figure 10:**
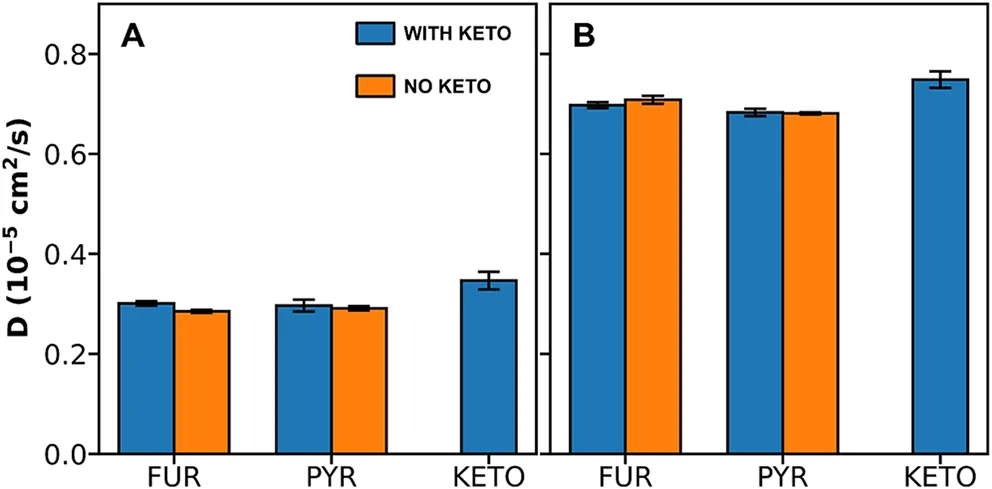
Diffusion coefficients of fructose tautomers in systems with and without KETO at (A) 1.85 M and (B) 0.28 M total fructose concentration. All the simulation data were obtained at 300 K. Corrections were not applied to account for the finite size of the simulation boxes. The system compositions are shown in Tables 2 and 3. The error bars represent the 95% confidence interval obtained using Student’s *t*-distribution.

The results so far suggest that aggregation of fructose molecules is the primary mechanism controlling molecular mobility. The aggregation between fructose is mediated by water molecules, as shown pictorially in the snapshots of Figures 4, 7, and 8. Because the presence of ions can affect the structure of water,^71^ it is possible that ions present in the aqueous systems affect aggregation, and therefore the diffusion coefficients. To test this possibility, MD simulations were conducted at NaCl concentrations of 0.075, 0.375, and 0.744 M. The results were compared to those obtained in the presence of NaCl in Figure S12. For this analysis, the fructose concentration was maintained at 0.28 and 1.85 M. In dilute fructose solutions (0.28 M), introducing NaCl induces a minor attenuation in the diffusion of fructose FUR and PYR. The differences are less pronounced at high fructose concentration (1.85 M). It was concluded that NaCl has little effect on the diffusion of fructose.

### 3.5 Mobility of Water in Aqueous Fructose Systems

The simulation snapshots shown in Figure 4 suggest that water molecules mobility is strongly influenced by the concentration-dependent organisation of fructose molecules. At low fructose concentrations, the sugar molecules are dispersed throughout the solution, allowing water molecules to diffuse relatively freely. At higher fructose concentrations, such as 4.85 M, fructose molecules aggregate into extended networks, which are expected to hinder water diffusion. This hypothesis was examined using both MD simulations and experimental PFG-NMR data.^5,29^ The resulting water diffusion coefficients are shown in Figure 11, while the corresponding PFG log plots are provided in Figure S8. Both computational and experimental results show the same trend, namely that the diffusion coefficient decreases monotonically with increasing fructose concentration. At low concentrations (<2 M), the experimental diffusion coefficients are slightly lower than the simulated values. This difference may be attributed to the solvent isotope effect, since D_2_O exhibits a higher viscosity than H_2_O and therefore gives rise to slower molecular diffusion. Using the Stokes-Einstein relationship, it can be estimated that the isotopic effect of changing H_2_O with D_2_O leads to a decrease in 15–20% of the expected diffusion coefficient. It should also be noted that the literature NMR data shown in Figure 2 were acquired at 298 K in H_2_O, whereas the new measurements reported here were performed at 301 K in D_2_O. The slightly higher temperature employed in the present study is expected to result in somewhat larger values of diffusion coefficients, which may contribute to the observed differences between the two experimental datasets. The increase in temperature could enhance the diffusion coefficient by ∼7%. Therefore, the combined effects of isotopic substitution and different temperature should decrease the diffusion coefficient by ∼10%. This qualitative analysis suggests that the literature NMR data are slower than expected based on the new NMR data collected in this work. To support the reliability of the new experiments, it is notable that at fructose concentrations higher than ∼2 M, the experimental NMR data are in strong agreement with the simulation results. At these conditions, as suggested by Figure 4, the aggregation between fructose molecules restricts the motion of water.

**Figure 11:**
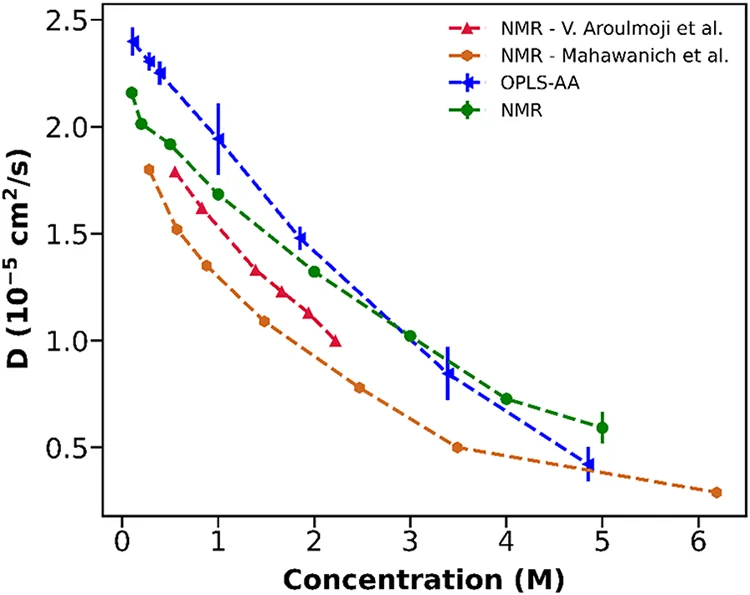
Water diffusion coefficients in aqueous solutions as a function of fructose concentration. Experimental NMR data obtained at 298 K are from the literature,^5,29^ as well as from the new PFG-NMR experiments conducted in deuterated water at 301 K for this work. Simulation data, obtained at 300 K, were extrapolated at the limit for an infinite simulation box size, as explained in the SI, implementing the OPLS-AA force fields. The error bars represent the 95% confidence interval obtained using Student’s *t*-distribution.

The effect of NaCl and the presence of the linear KETO tautomer on water diffusion were also examined using molecular simulations implementing the validated force fields. The results are shown in Figure S13. At both low and high fructose concentrations, neither NaCl nor KETO affect water dynamics. The limited influence of NaCl can be attributed to the relatively low NaCl concentrations employed in this study (maximum 0.744 M). Harris et al.^72^ reported a noticeable reduction in water diffusivity only manifests at salt concentrations exceeding 2 M. Thus, the salt concentration considered here is likely too low to perturb water mobility in aqueous fructose solutions substantially.

## 4 Conclusions

Fructose is an abundant and versatile biomass-derived molecule that is widely used in the food industry and is being explored as a renewable feedstock for producing chemicals and fuels. The rational design of fructose conversion processes requires a quantitative understanding of its transport properties in aqueous systems, particularly as a function of concentration and of solution composition. In this study, a combined MD and PFG-NMR approach was employed to establish structure-transport relationships in aqueous fructose solutions. New PFG-NMR measurements were conducted in deuterated water to enhance the separation of fructose and water signals and provide a rigorous benchmark for the simulations.

Among the force fields evaluated, OPLS-AA was found to provide the best agreement with the experimental density and diffusion data over a broad concentration range. Upon validation, molecular simulations were employed to gain molecular insights into the structure of such aqueous systems. The simulation trajectories revealed that the two dominant cyclic fructose tautomers, β-d-fructofuranose and β-d-fructopyranose, exhibit similar mobilities in aqueous solution, whereas the minor acyclic keto form diffuses somewhat faster. The addition of NaCl at low concentrations had a negligible effect on fructose transport. The combined experimental and computational analyses further showed that both fructose and water diffusion coefficients decrease with increasing fructose concentration. Structural analyses revealed that this behaviour originates from the progressive formation of fructose-rich aggregates and clusters, which increasingly restrict molecular motion. Hydrogen-bond analyses demonstrated a transition from predominantly fructose–water interactions at low fructose concentrations to increasingly persistent fructose–fructose association at high concentrations. The formation of long-lived solute–solute hydrogen-bond networks provides a molecular explanation for the observed reduction in diffusivity and establishes a clear structure–transport relationship for aqueous fructose solutions. While these results present a clear structure-transport relation for aqueous fructose, to be applicable to catalytic processes it is necessary to extend these studies to systems containing catalysts at experimentally relevant conditions.

## Supplementary Material



## Notes

Mean square displacement (MSD) of fructose molecules obtained from simulations conducted implementing OPLS-AA, GLYCAM06, and CHARMM force fields; finite-size effect corrections for the simulated diffusion coefficient of fructose and water molecules at various concentrations for simulations conducted implementing OPLS-AA and GLYCAM06; comparison of fructose solution densities obtained from simulations and experiments at various concentrations; diffusion coefficients of fructose and water before finite-size corrections, obtained from simulations and experiments; experimental PFG-NMR log-attenuation plots of water and fructose in D_2_O; diffusion coefficients of fructose tautomers at various concentrations; MSDs of fructose tautomers at various salt concentrations, with and without the presence of the KETO configuration; simulated diffusion coefficients of fructose tautomers and water at various salt concentrations, with and without the presence of the KETO configuration (PDF)
