# Supplementary material for: Structure-Transport Relationships in Aqueous Fructose Solutions: A Combined Molecular Dynamics and NMR Diffusion Study
Source: J Phys Chem B. 2026 Sep 16;130(39):10298–309. doi: 10.1021/acs.jpcb.6c04242 (PMC13637019; doi:10.1021/acs.jpcb.6c04242)
Supplement: Supplementary file 1 [file jp6c04242_si_001.pdf]

Structure - Transport Relationships in Aqueous Fructose Solutions: A Combined Molecular Dynamics and NMR Diffusion Study

San Vo Vinh Phan [a], Xinyue Zhou [b], Gabriel D. Barbosa [a], Tran Thi Bao Le [a], Christopher M.A Parlett [b], Xiaolei Fan [b,*], Carmine D'Agostino[b,c,*] and Alberto Striolo [a,d,*]

[a] *School of Sustainable Chemical, Biological and Materials Engineering, The University of Oklahoma, 100 E. Boyd Street, Norman, OK 73019, United States*

[b] *Department of Chemical Engineering, The University of Manchester Oxford Road, Manchester M13 9PL, UK*

[c] *Dipartimento di Ingegneria Civile, Chimica, Ambientale e dei Materiali (DICAM), Alma Mater Studiorum – Università di Bologna, Via Terracini, 28, 40131 Bologna, Italy*

[d] *Materials Science and Engineering Program, The University of Oklahoma, Norman, OK 73019, USA*

SUPPLEMENTARY INFORMATION

* Corresponding Authors:

Xiaolei Fan: *Xiaolei.fan@manchester.ac.uk*

Carmine D'Agostino: *carmine.dagostino@manchester.ac.uk*

Alberto Striolo: *astriolo@ou.edu*

This Supplementary Information contains simulation and experimental data supporting the results discussed in the main text. The results are organized in the following figures:

Figure S1. The mean square displacement of fructose molecules obtained conducting molecular dynamics simulations at various fructose concentrations. The results are shown for the different force fields implemented: A. OPLS-AA, B. GLYCAM06, and C. CHARMM. The simulations were conducted at T=300 K and P=1 atm. The error bars represent the 95% confidence interval obtained using Student's t-distribution.

Figure S2. Finite-size effect correction for the simulated diffusion coefficient of fructose as obtained using the OPLS-AA force field at different concentrations of fructose in water: (A) 0.11 M, (B) 0.28 M, (C) 0.39 M, (D) 1.00 M, (E) 1.85 M, (F) 3.39 M, and (G) 4.85 M. The simulation data were obtained at 300 K and 1 atm. The statistical uncertainties were obtained from four independent simulations for each box size. The error bars represent the 95% confidence interval obtained using Student's t-distribution.

Figure S3. Finite-size effect correction for the diffusion of water obtained using the OPLS-AA force field at different fructose concentrations: (A) 0.11 M, (B) 0.28 M, (C) 0.39 M, (D) 1.00 M, (E) 1.85 M, (F) 3.39 M, and (G) 4.85 M. The simulation data were obtained at 300 K and 1 atm. The statistical uncertainties were obtained from four independent simulations for each box size. The error bars represent the 95% confidence interval obtained using Student's t-distribution.

Figure S4. Same as Figure S2, using the GLYCAM06 force field.

Figure S5. Same as Figure S3, using the GLYCAM06 force field.

Figure S6. Density of fructose solutions as a function of fructose concentration. Results are obtained from molecular dynamics simulations using three different force fields at 300K and 1 atm. Experimental results were obtained in H_2O and D_2O at ambient conditions (298 K, 1 atm). The error bars represent the 95% confidence interval obtained using Student's t-distribution.

Figure S7. Simulated diffusion coefficient of fructose (A) and water (B) at various fructose concentrations in water. Results are obtained implementing different force fields. The simulations were

conducted at 300 K and 1 atm. Experimental NMR data, obtained at 298 K, are from the literature.[1, 2] The error bars represent the 95% confidence interval obtained using Student's t-distribution.

Figure S8. Experimental PFG-NMR log attenuation plots of residual proton-containing water and fructose in D_2O obtained at various fructose concentrations at 301K and 1 atm. The results for water were extracted from the residual water signal at ~4.6 ppm. Fitted blue and red continuous lines for water and fructose are included to guide the eyes, respectively.

Figure S9. Simulated diffusion coefficients obtained for two fructose tautomers. Simulation data are extrapolated at the limit for an infinite simulation box size. The simulations were conducted implementing the OPLS-AA force fields at 300 K. The error bars represent the 95% confidence interval obtained using Student's t-distribution.

Figure S10. The mean square displacement of fructose tautomers. From left to right, the panels are obtained at increasing NaCl concentration: (A) 0 M, (B) 0.075 M, (C) 0.375 M, (D) 0.744 M. The concentration of fructose is maintained fixed at 0.3 M in all simulations. The OPLS-AA force field is used for simulation. The upper panels are for the systems that contains KETO, the lower panels are for the system with no KETO. The error bars represent the 95% confidence interval obtained using Student's t-distribution.

Figure S11. Same as Figure S10, with the concentration of fructose fixed at 1.85 M.

Figure S12. Simulated diffusion coefficients of each fructose tautomer in systems with and without KETO at different NaCl concentrations. Panels (A–C) correspond to systems with a fructose concentration of 0.3 M and NaCl concentrations of 0.075 M, 0.375 M, and 0.744 M, respectively, while panels (D–F) correspond to systems with a fructose concentration of 1.85 M at the same NaCl concentrations. All data were obtained at 300 K. The error bars represent the 95% confidence interval obtained using Student's t-distribution.

Figure S13. Diffusion coefficients of water in systems with KETO as a function of the NaCl concentration. The fructose concentrations considered are 0.28 M and 1.85 M. The simulation data were obtained at 300 K. Experimental NMR data collected at 298K for a solution with salt only are

taken from the literature.[3] The error bars represent the 95% confidence interval obtained using Student's t-distribution.

Table S1. Simulated diffusion coefficients obtained using the OPLS-AA force field at 300 K with 95% confidence interval (obtained using Student's t-distribution) provided.

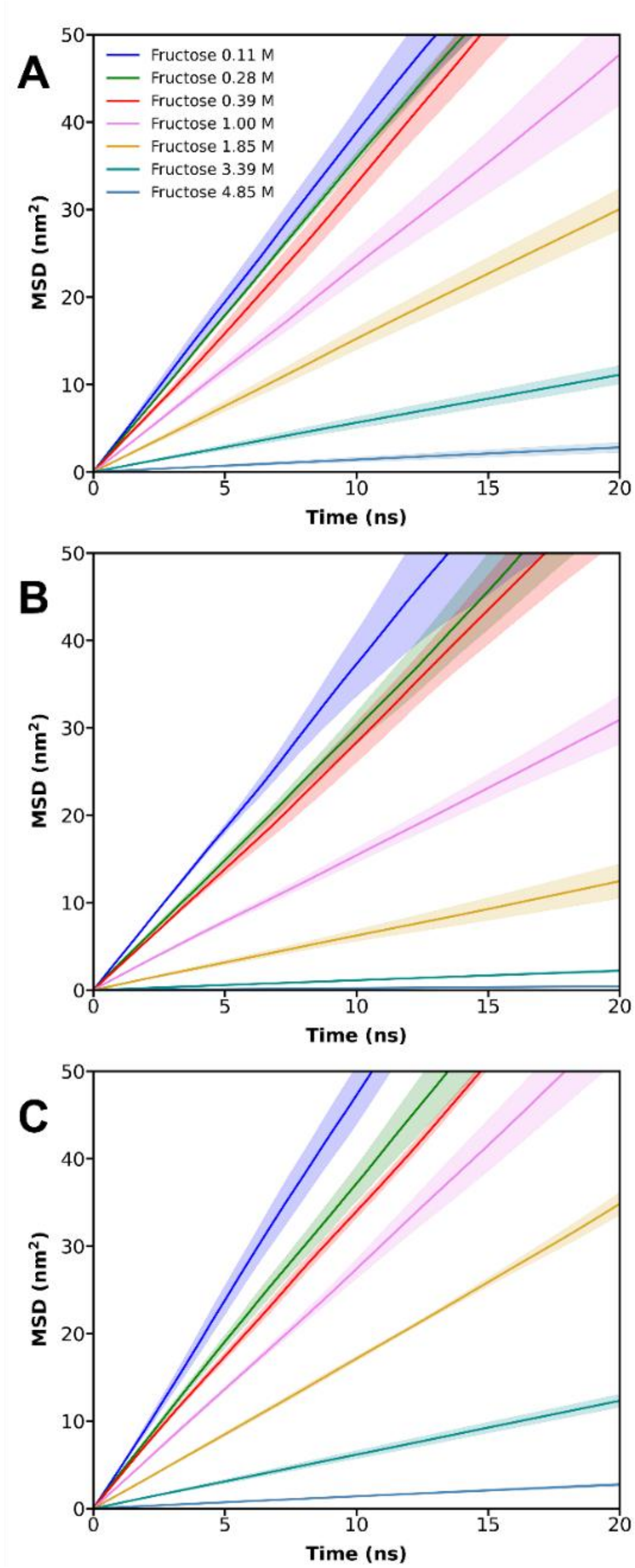

Figure S1. The mean square displacement of fructose molecules obtained conducting molecular dynamics simulations at various fructose concentrations. The results are shown for the different force fields implemented: A. OPLS-AA, B. GLYCAM06, and C. CHARMM. The simulations were conducted at T=300 K and P=1 atm. The error bars represent the 95% confidence interval obtained using Student's t-distribution.

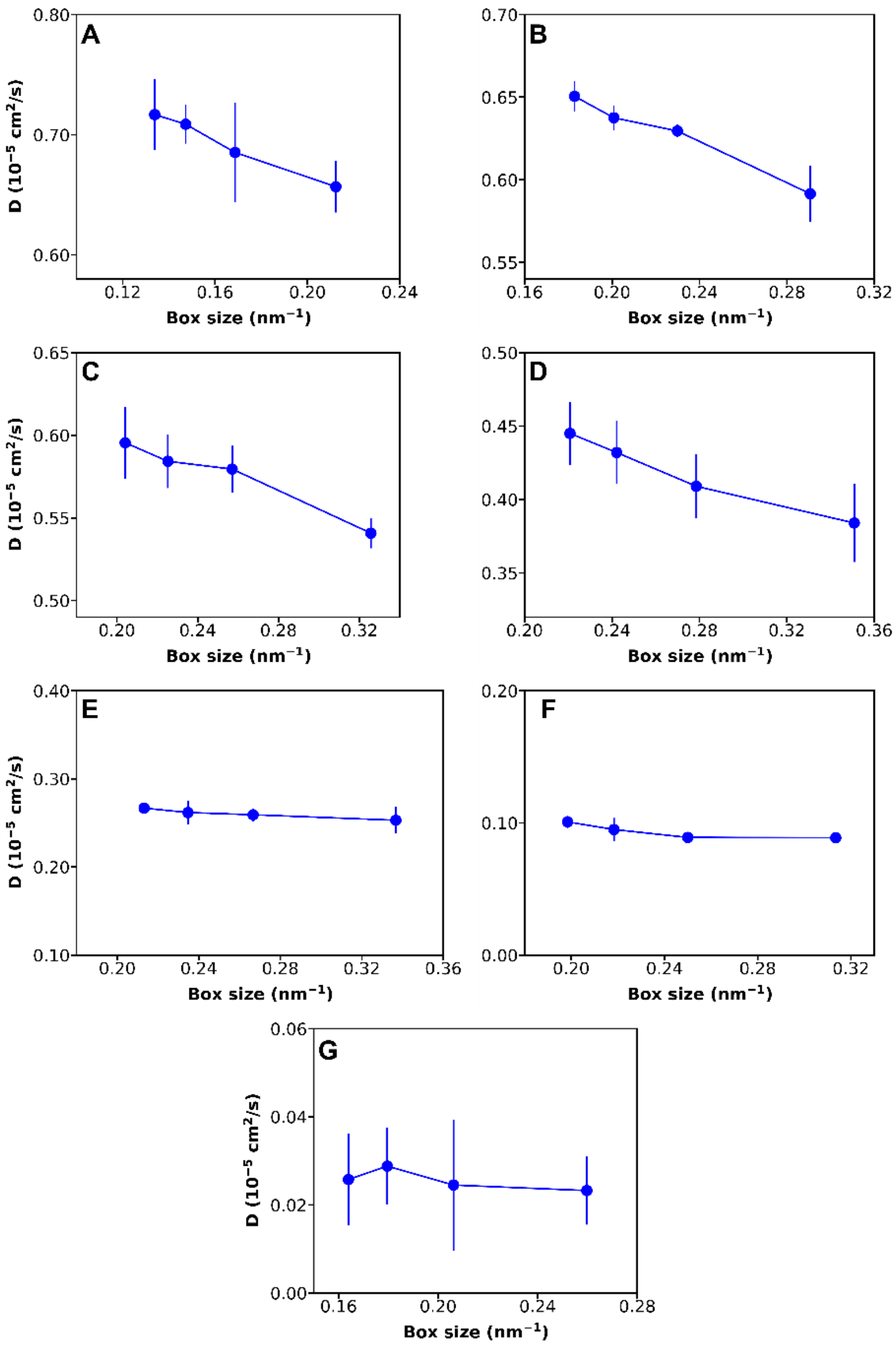

Figure S2. Finite-size effect correction for the simulated diffusion coefficient of fructose as obtained using the OPLS-AA force field at different concentrations of fructose in water: (A) 0.11 M, (B) 0.28 M, (C) 0.39 M, (D) 1.00 M, (E) 1.85 M, (F) 3.39 M, and (G) 4.85 M. The simulation data were obtained at 300 K and 1 atm. The statistical uncertainties were obtained from four independent simulations for each box size. The error bars represent the 95% confidence interval obtained using Student's t-distribution.

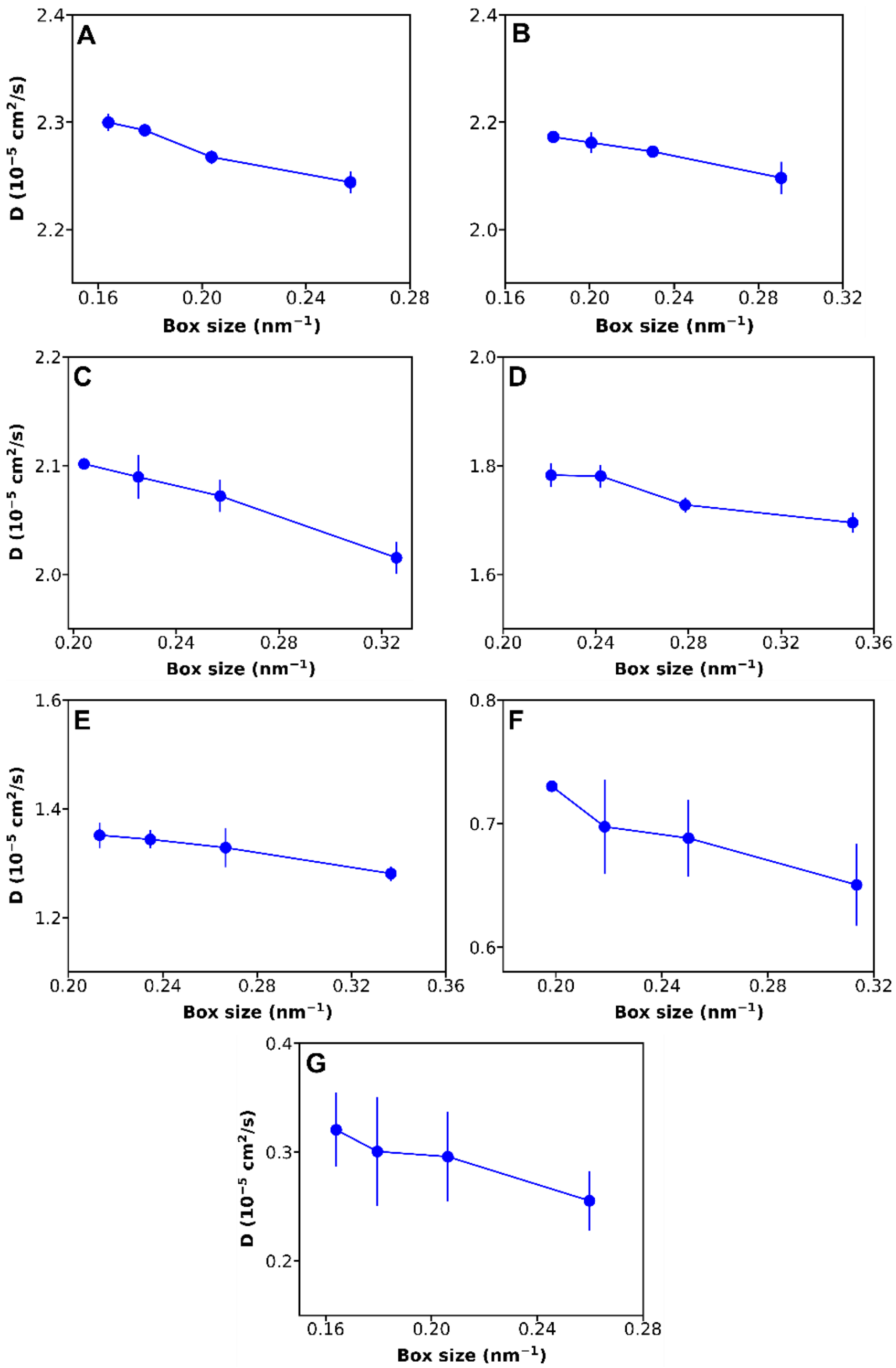

Figure S3. Finite-size effect correction for the diffusion of water obtained using the OPLS-AA force field at different fructose concentrations: (A) 0.11 M, (B) 0.28 M, (C) 0.39 M, (D) 1.00 M, (E) 1.85 M, (F) 3.39 M, and (G) 4.85 M. The simulation data were obtained at 300 K and 1 atm. The statistical uncertainties were obtained from four independent simulations for each box size. The error bars represent the 95% confidence interval obtained using Student's t-distribution.

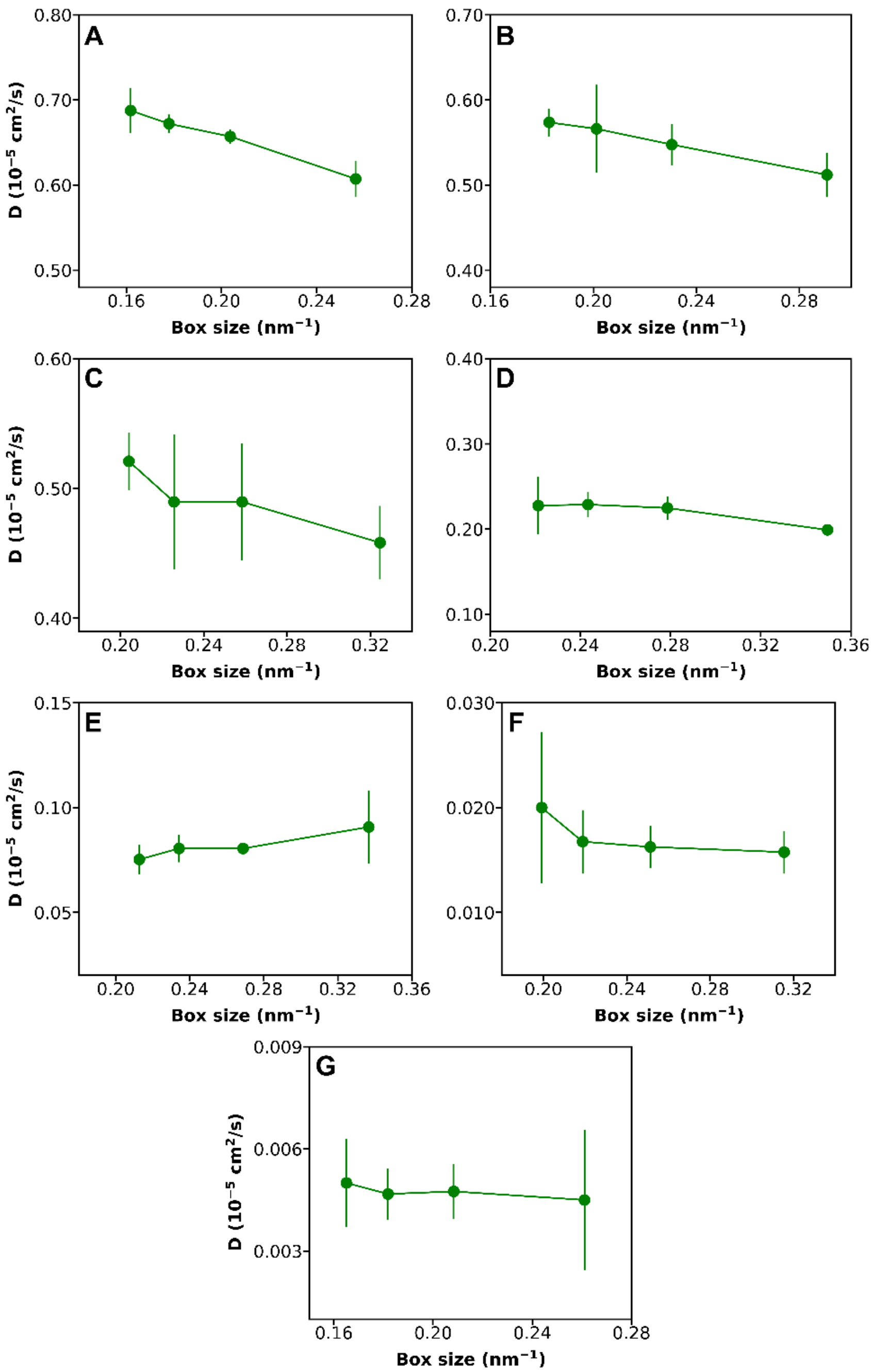

Figure S4. Same as Figure S2, using the GLYCAM06 force field.

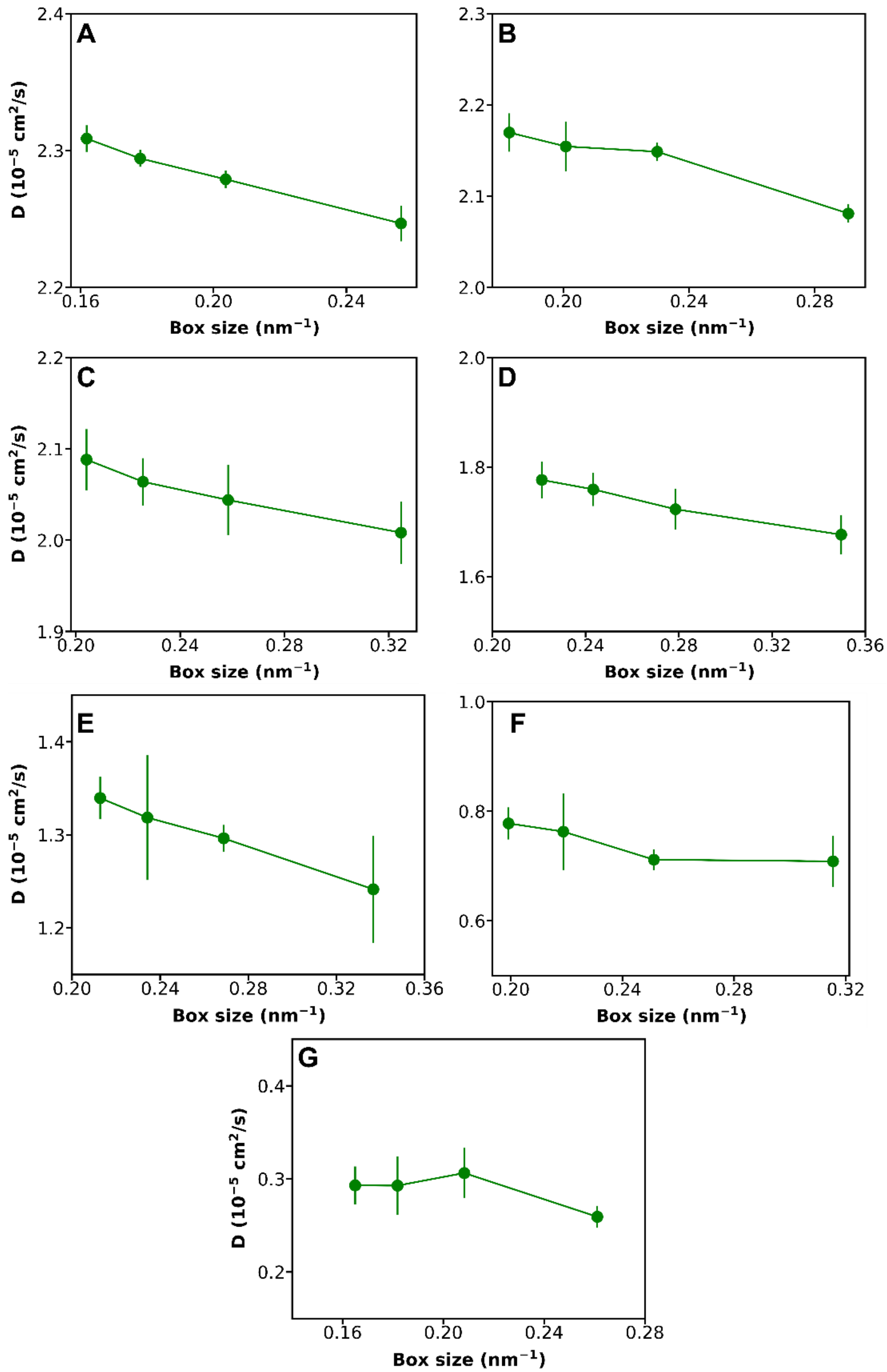

Figure S5. Same as Figure S3, using the GLYCAM06 force field.

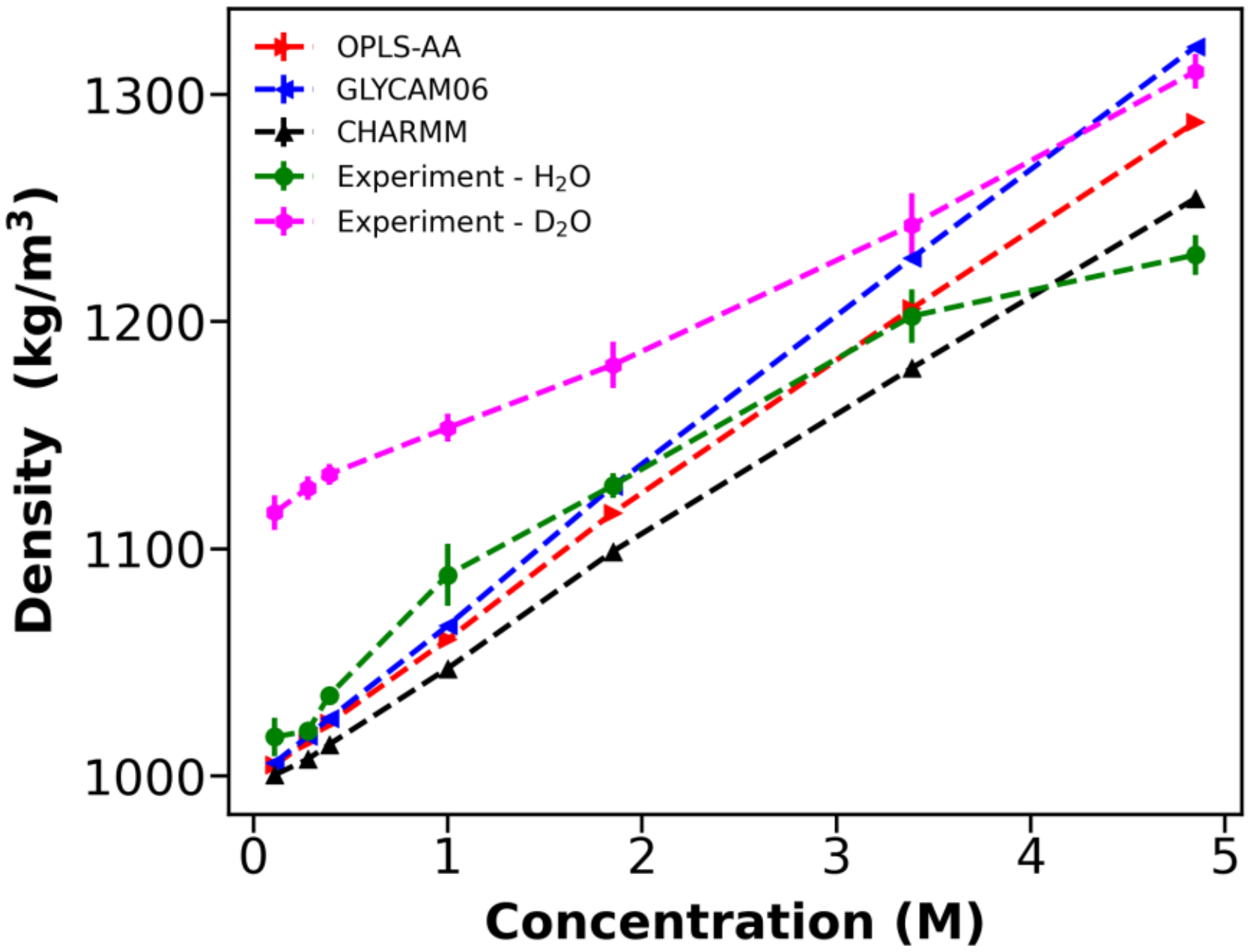

Figure S6. Density of fructose solutions as a function of fructose concentration. Results are obtained from molecular dynamics simulations using three different force fields at 300K and 1 atm. Experimental results were obtained in H_2O and D_2O at ambient conditions (298 K, 1 atm). The error bars represent the 95% confidence interval obtained using Student's t-distribution.

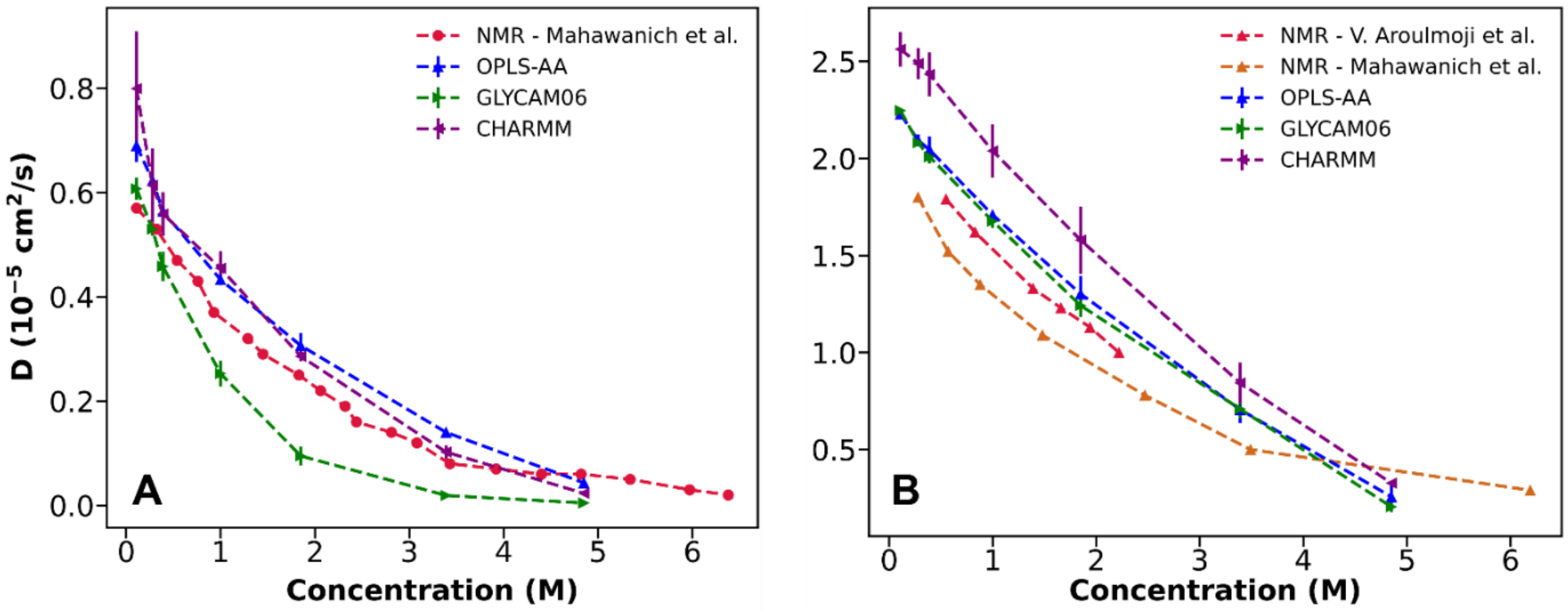

Figure S7. Simulated diffusion coefficient of fructose (A) and water (B) at various fructose concentrations in water. Results are obtained using different force fields. The simulations were conducted at 300 K and 1 atm. Experimental NMR data, obtained at 298 K, are from the literature.[1, 2] The error bars represent the 95% confidence interval obtained using Student's t-distribution.

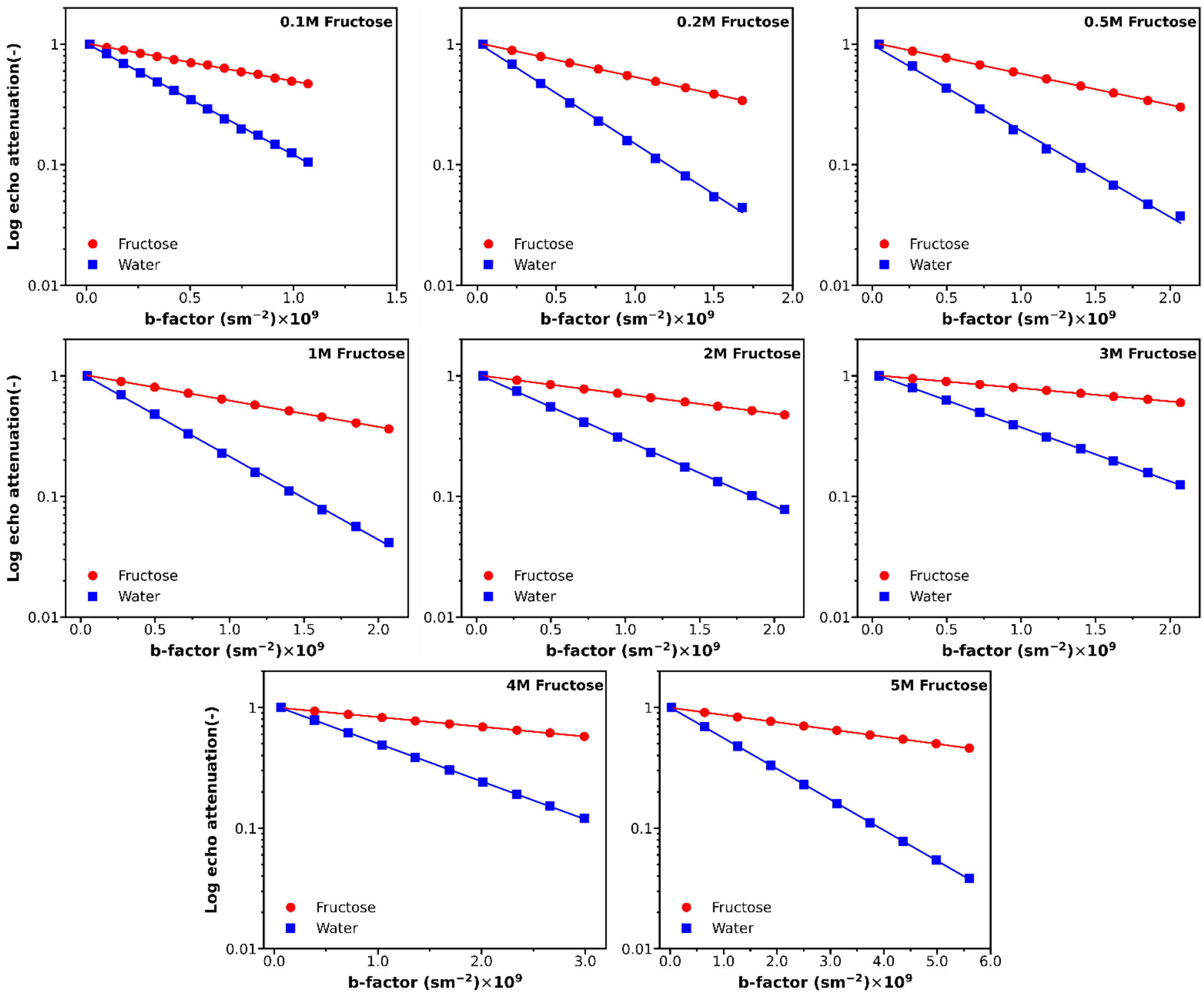

Figure S8. Experimental PFG-NMR log attenuation plots of residual proton-containing water and fructose in D_2O obtained at various fructose concentrations at 301K and 1 atm. The results for water were extracted from the residual water signal at ~4.6 ppm. Fitted blue and red continuous lines for water and fructose are included to guide the eyes, respectively.

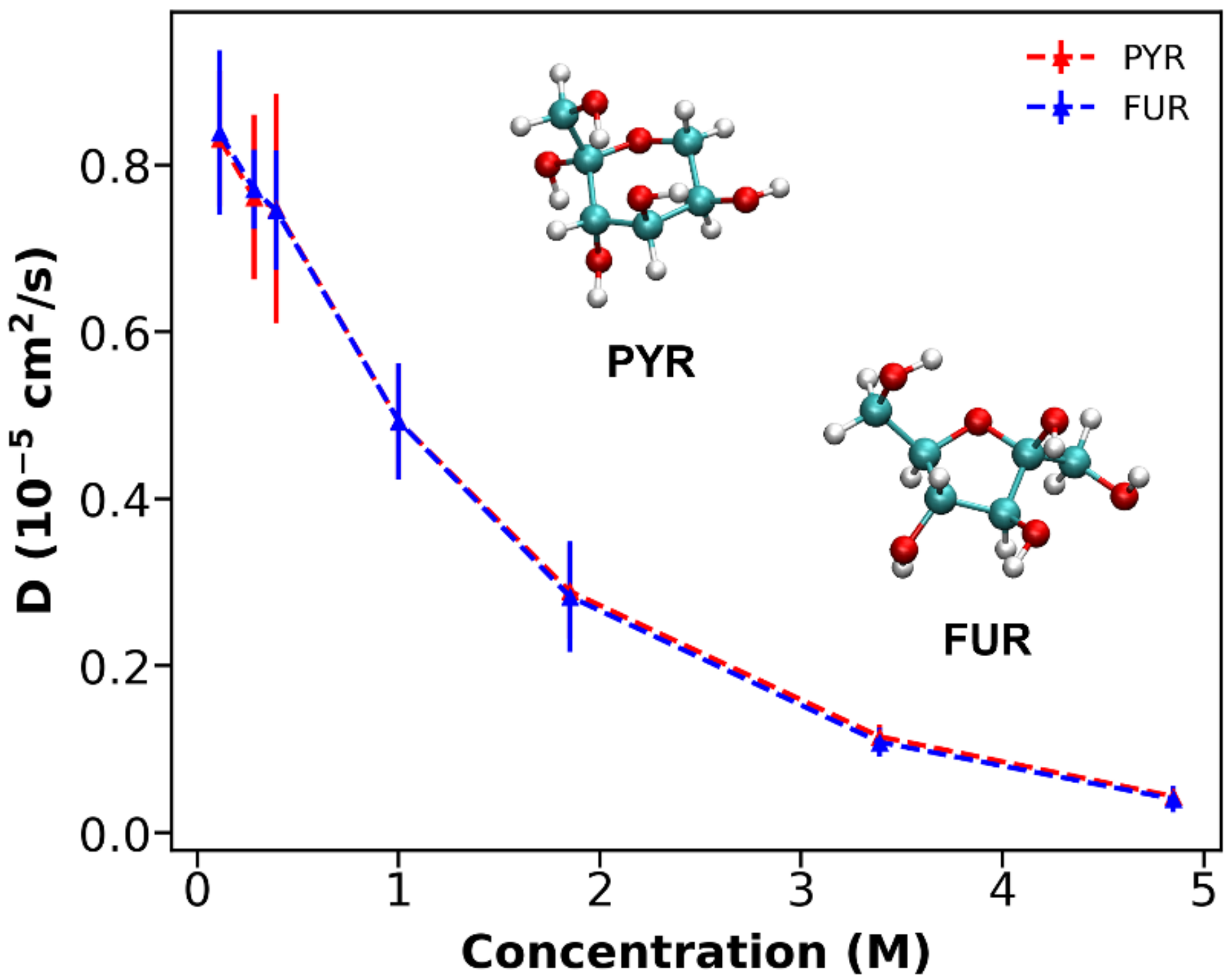

Figure S9. Simulated diffusion coefficients obtained for two fructose tautomers. Simulation data are extrapolated at the limit for an infinite simulation box size. The simulations were conducted implementing the OPLS-AA force fields at 300 K. The error bars represent the 95% confidence interval obtained using Student's t-distribution.

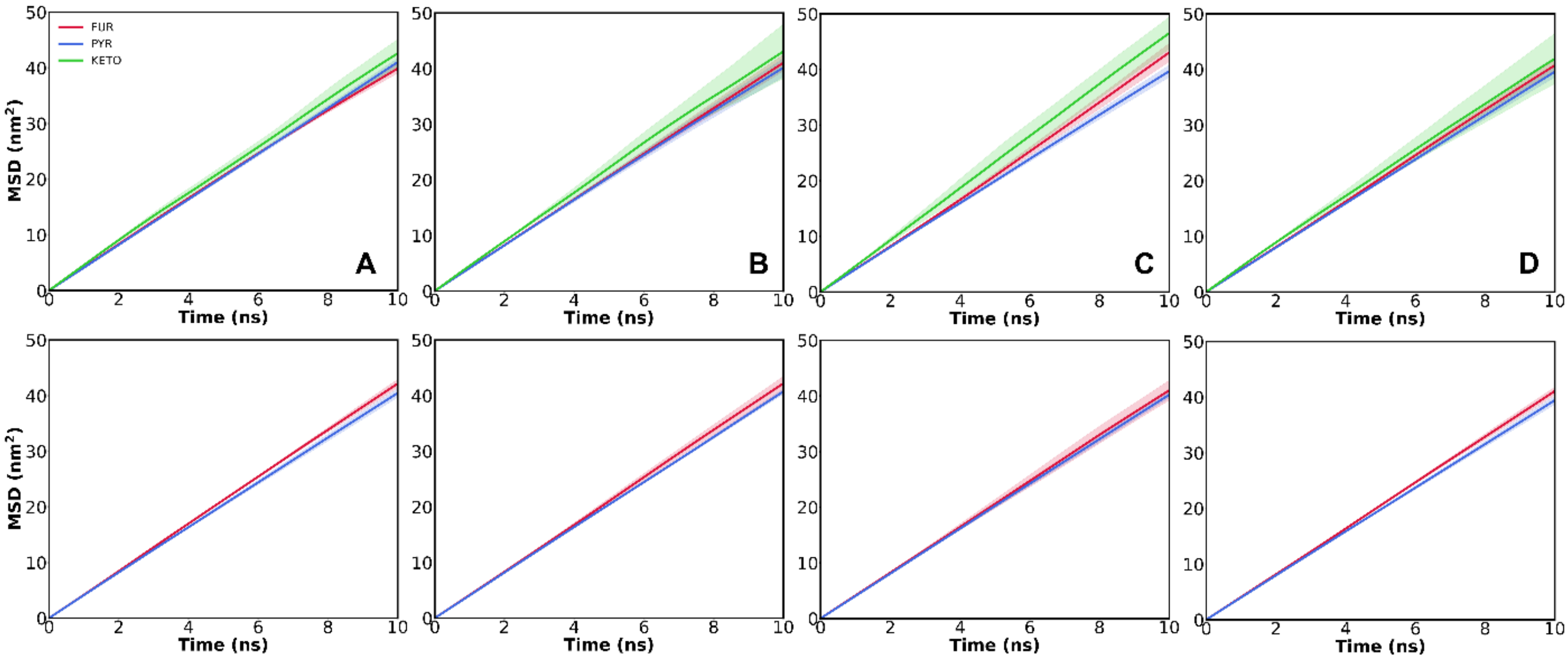

Figure S10. The mean square displacement of fructose tautomers. From left to right, the panels are obtained at increasing NaCl concentration: (A) 0 M, (B) 0.075 M, (C) 0.375 M, (D) 0.744 M. The concentration of fructose is maintained fixed at 0.28 M in all simulations. The OPLS-AA force field is used for simulation. The upper panels are for the systems that contains KETO, the lower panels are for the system with no KETO. The error bars represent the 95% confidence interval obtained using Student's t-distribution.

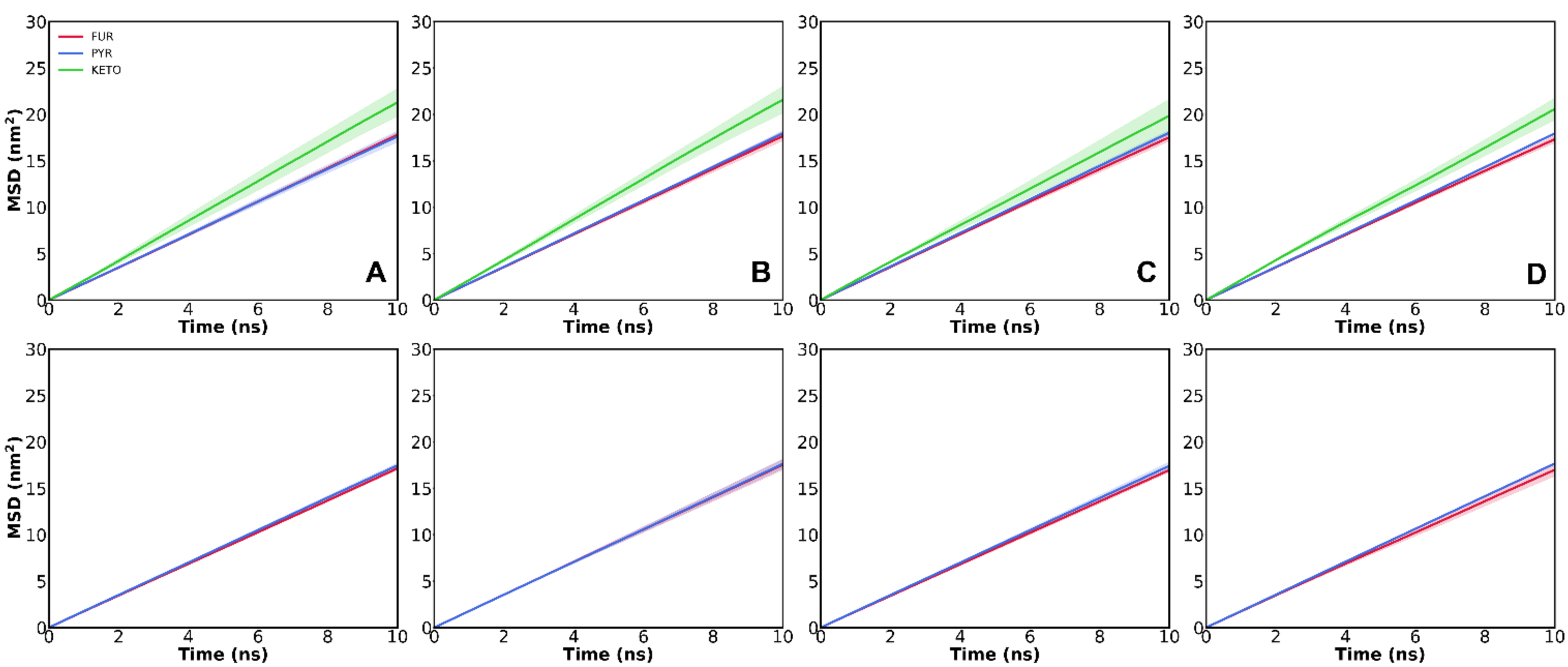

Figure S11. Same as Figure S10, with the concentration of fructose fixed at 1.85 M.

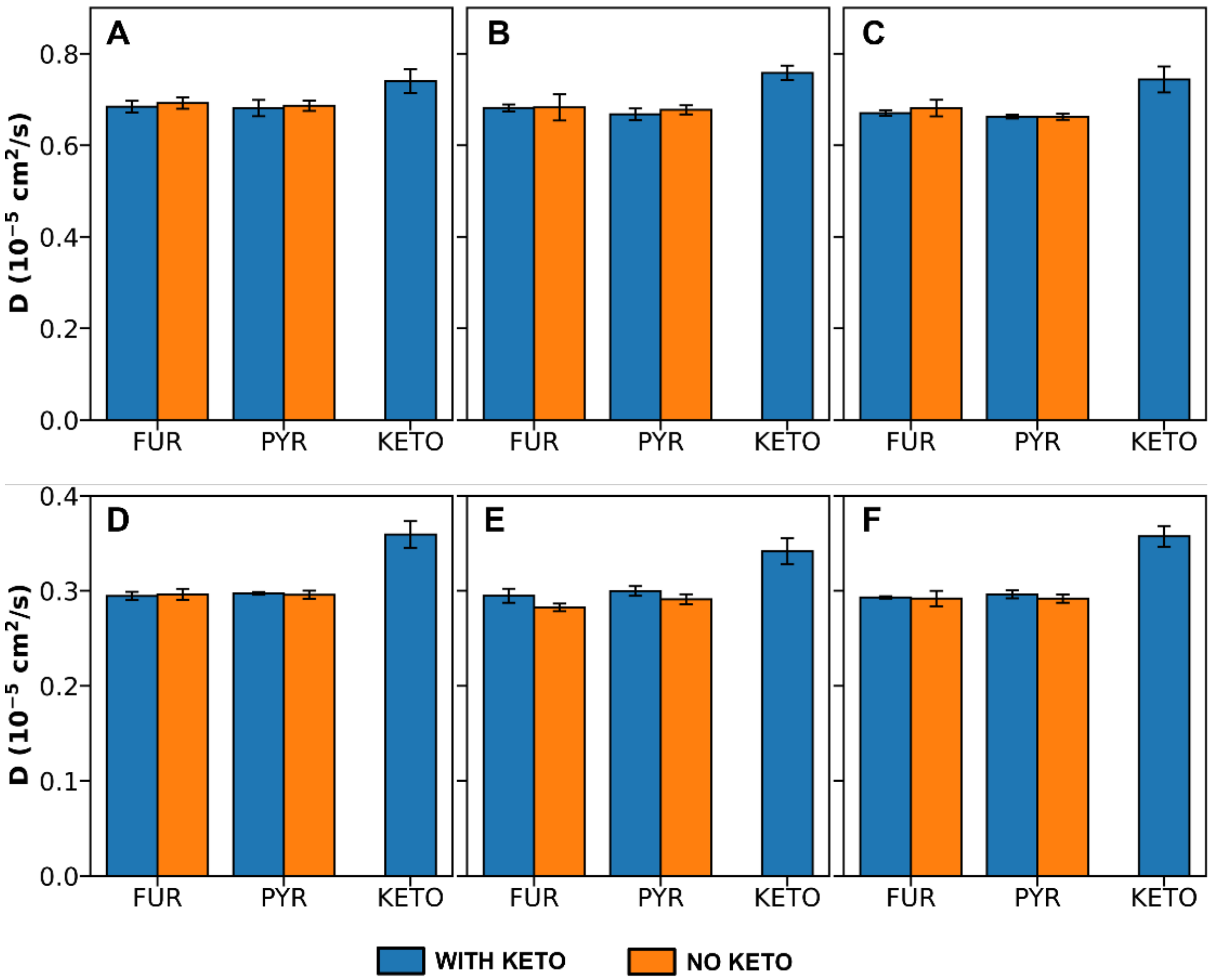

Figure S12. Simulated diffusion coefficients of each fructose tautomer in systems with and without KETO at different NaCl concentrations. Panels (A–C) correspond to systems with a fructose concentration of 0.28 M and NaCl concentrations of 0.075 M, 0.375 M, and 0.744 M, respectively, while panels (D–F) correspond to systems with a fructose concentration of 1.85 M at the same NaCl concentrations. All data were obtained at 300 K. The error bars represent the 95% confidence interval obtained using Student's t-distribution.

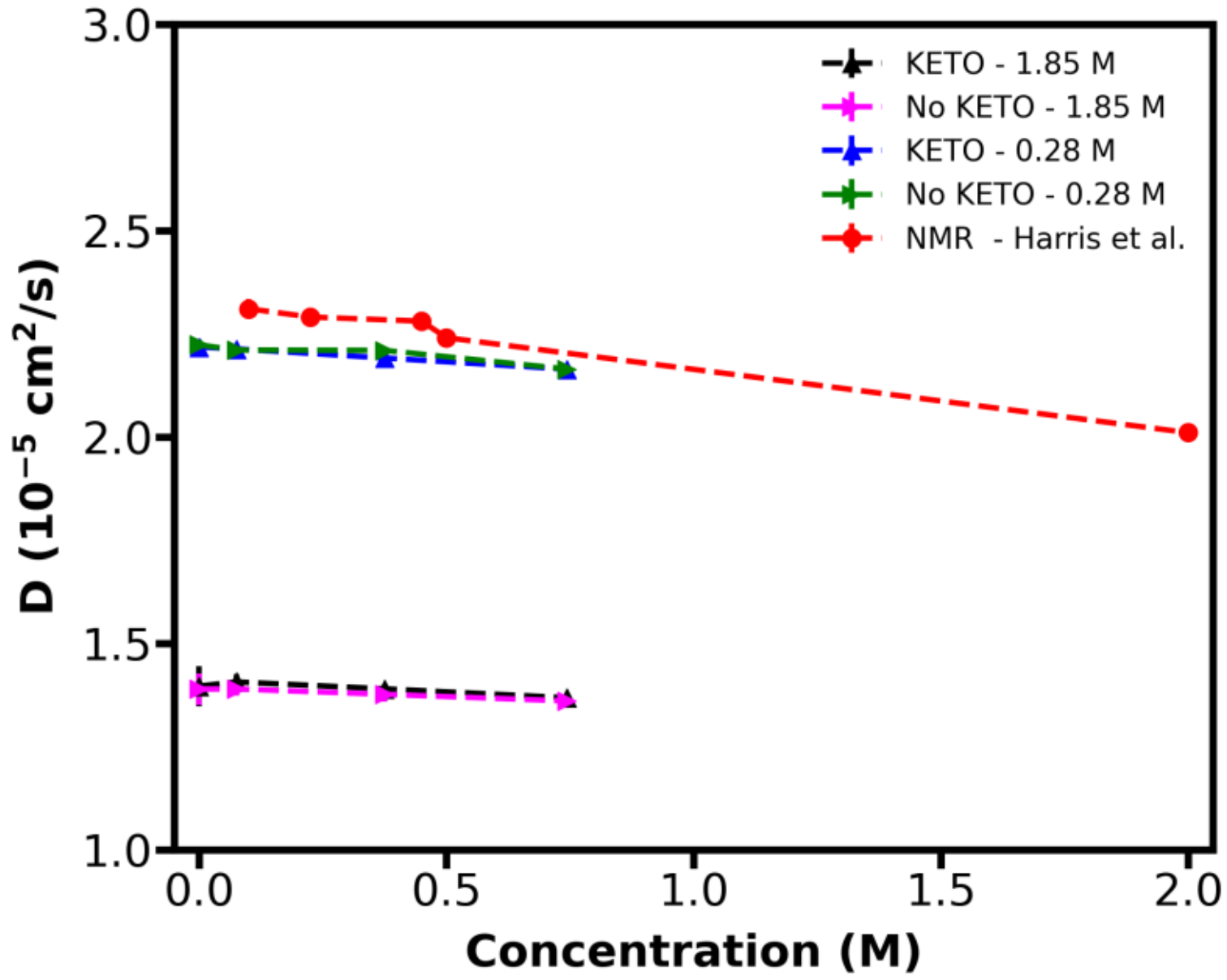

Figure S13. Diffusion coefficients of water in systems with KETO as a function of the NaCl concentration. The fructose concentrations considered are 0.28 M and 1.85 M. The simulation data were obtained at 300 K. Experimental NMR data collected at 298K for a solution with salt only are taken from the literature.[3] The error bars represent the 95% confidence interval obtained using Student's t-distribution.

Table S1. Simulated diffusion coefficients for fructose in water as obtained using the OPLS-AA force field at 300 K with 95% confidence interval (obtained using Student's t-distribution) provided.

Fructose Concentration (M)	D (10^{-5} cm^2/s)
0.11	0.821 ± 0.044
0.28	0.744 ± 0.067
0.39	0.683 ± 0.062
1	0.544 ± 0.062
1.85	0.287 ± 0.018
3.39	0.117 ± 0.046
4.85	0.034 ± 0.024

References

(1) Mahawanich, T.; Schmidt, S. J. Molecular mobility and the perceived sweetness of sucrose, fructose, and glucose solutions. *Food Chemistry* **2004**, *84* (2), 169-179. DOI: 10.1016/s0308-8146(03)00197-3.

(2) Aroulmoji, V.; Mathlouthi, M.; Feruglio, L.; Murano, E.; Grassi, M. Hydration properties and proton exchange in aqueous sugar solutions studied by time domain nuclear magnetic resonance. *Food chemistry* **2012**, *132* (4), 1644-1650.

(3) Harris, K. R.; Mills, R.; Back, P. J.; Webster, D. S. An improved NMR spin-echo apparatus for the measurement of self-diffusion coefficients: The diffusion of water in aqueous electrolyte solutions. *Journal of Magnetic Resonance (1969)* **1978**, *29* (3), 473-482. DOI: https://doi.org/10.1016/0022-2364(78)90005-7.
